# Quantifying the influence of 3d–4s mixing on linearly coordinated metal-ions by L_2,3_-edge XAS and XMCD†

**DOI:** 10.1039/d3sc06308a

**Published:** 2023-12-26

**Authors:** Myron S. Huzan, Timothy G. Burrow, Manuel Fix, Franziska A. Breitner, Sut Kei Chong, Peter Bencok, Matteo Aramini, Anton Jesche, Michael L. Baker

**Affiliations:** a Department of Chemistry, The University of Manchester Manchester M13 9PL UK michael.baker@manchester.ac.uk; b The University of Manchester at Harwell, Diamond Light Source, Harwell Campus OX11 0DE UK; c EP VI, Center for Electronic Correlations and Magnetism, Institute of Physics, University of Augsburg D-86159 Augsburg Germany; d Diamond Light Source, Harwell Science and Innovation Campus Chilton, Didcot OX11 0DE UK

## Abstract

The mixing valence d and s orbitals are predicted to strongly influence the electronic structure of linearly coordinated molecules, including transition metals, lanthanides and actinides. In specific cases, novel magnetic properties, such as single-ion magnetic coercivity or long spin decoherence times, ensue. Inspired by how the local coordination symmetry can engender such novel phenomena, in this study, we focus our attention on dopants (Mn, Fe, Co, Ni, Cu) in lithium nitride to accept innovation from molecular magnetism in a high symmetry *P*6/*mmm* solid-state crystal. The linear coordination environment results in strong 3d–4s mixing, proving to be an ideal series to investigate the role of d–s mixing and bonding on electronic structure and magnetism. It is shown that L_2,3_-edge XAS can be applied to experimentally identify the presence of 3d–4s mixing and the influence this has on the ligand-field splitting. XMCD specifies how spin–orbit coupling is affected. The combined spectroscopies are analysed to determine the effect of 4s mixing with support from *ab initio* calculations. The results provide new insight of relevance to future applications, including quantum information processing and the sustainable replacement of rare earths in magnets.

## Abstract 2

X-ray absorption spectroscopy and dichroism are applied to probe the effect of 3d–4s mixing on electronic structure and magnetism for linearly coordinated Mn, Fe, Co, Ni and Cu.

## Graphical abstract

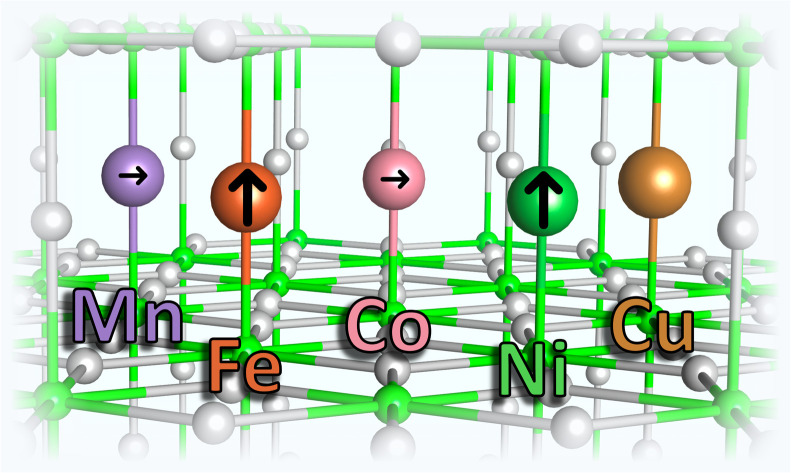

## 1 Introduction

The selection of solid-state compounds with local coordination environments that mimic novel molecular complexes provides a promising route to accept innovation from molecular magnetism at a dopant site while maintaining a rigid solid-state extended coordination environment to reduce spin-phonon coupling and maintain rigorous local coordination symmetry. In this light, we look towards the growing interest in low coordination complexes within the field of molecular magnetism due to exceptional energy barriers to magnetisation reversal^1,2^ and high-frequency quantum clock transitions.^3^ It has been shown that molecular-based linearly coordinated transition metal-ions can facilitate an unquenched orbital angular momentum (*L*) unperturbed by Jahn–Teller distortions.^4^ Prominent linear single-ion magnets (SIMs) include divalent [Co(C(SiMe_3_)_3_)_2_]^2−^ and monovalent [Fe(C(SiMe_3_)_3_)_2_]^1−^ which exhibit pronounced magnetic remanence and slow magnetic relaxation. The former exhibits a non-Aufbau ground-state with maximal orbital angular momentum, *L* = 3, resulting from ligand-field energy stabilisation competing with interelectronic repulsions.

In a previous study, we applied single-crystal K-edge X-ray absorption near-edge structure and extended X-ray absorption fine structure to determine that iron doped in lithium nitride (Li_2_(Li_1−*x*_Fe_*x*_)N) is mono-valent, clean of stoichiometric vacancies where Fe sites are geometrically equivalent, linearly coordinated, occupying a *D*_6h_ symmetry pocket.^5,6^ The local geometric structure and oxidation state of Fe sites were found not to vary as a function of doping concentration (*x*) and variable temperature L_2,3_-edge XAS measurements showed that Li_2_(Li_1−*x*_Fe_*x*_)N exhibits a large spin reversal energy barrier, *U*_eff_ ≈ 33 meV.^5,7,8^ This energy barrier is comparable to [Fe(C(SiMe_3_)_3_)_2_]^1−^, however, the magnetisation relaxation time at low temperatures (*τ*) is many orders of magnitude longer in Li_2_(Li_1−*x*_Fe_*x*_)N, *τ* ∼ 10^4^ s (ref. 8) than [Fe(C(SiMe_3_)_3_)_2_]^−^ where *τ* ∼ 10^−2^ s. In this study, we conduct a detailed investigation of transition metal ions (TM) = Mn, Fe, Co, Ni and Cu doped in Li_2_(Li_1−*x*_TM_*x*_)N to investigate the role of metal-ion 3d–4s mixing and metal–ligand covalency on electronic structure and magnetism.

**Fig. 1:**
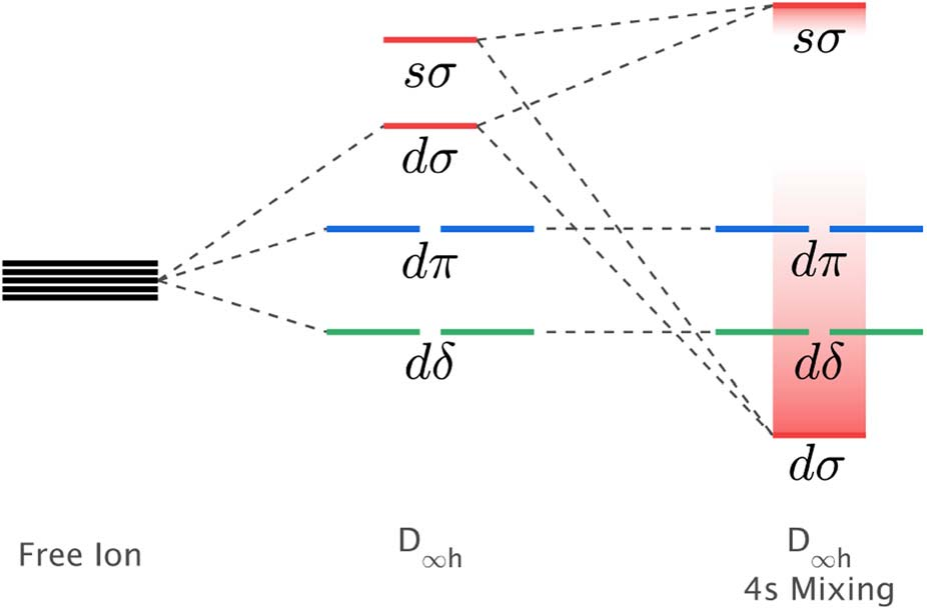
Schematic showing the influence of d–s orbital mixing on the energy of the d-orbitals for linearly coordinated transition metal ions.

The mixing of d–s metal-ion orbital character and related energy stabilisation of the d_*z*^2^_ orbital (Fig. 1) is of general importance to fundamental understandings of electronic structure. Both ligand-field theories^9–11^ and *ab initio* calculations^12,13^ predict d–s orbital mixing for metal-ions spanning the periodic table from transition metals, to lanthanides^14,15^ and actinides.^12,16^ Electron paramagnetic resonance (EPR) can be applied for Kramers ions to quantify d–s orbital mixing *via* the hyperfine effect. Enhanced hyperfine interactions due to d–s orbital mixing have been shown to result in avoided state crossings in applied magnetic fields with enhanced phase time memory (quantum clock transitions) of relevance to quantum computing.^3,17^ However, evidencing d–s mixing by EPR requires a d–s hybridised orbital to be singly occupied. This specificity limits experimental quantification of d–s mixing. Consequently, most reported insights derive from calculations, including complete active space self-consistent field (CASSCF) and density functional theory (DFT).^18^ In this work, we identify that X-ray absorption spectroscopy (XAS) can be applied to evaluate the impact of d–s mixing on electronic structure and magnetism. To identify how the linear ligand-field, including d–s mixing, relates to spin–orbit coupling, the XAS studies are combined with X-ray magnetic circular dichroism (XMCD). The results with accompanying theoretical calculations are interpreted to obtain insights into the superior magnetic properties exhibited for Li_2_(Li_1−*x*_Fe_*x*_)N and Li_2_(Li_1−*x*_Ni_*x*_)N relative to other linearly coordinated single-ion magnets.

## 2 Methods

### 2.1 Preparation of transition metal doped Li_3_N

Synthesis of Li_2_(Li_1−*x*_Mn_*x*_)N (Mn), Li_2_(Li_1−*x*_Fe_*x*_)N (Fe), Li_2_(Li_1−*x*_Co_*x*_)N (Co), Li_2_(Li_1−*x*_Ni_*x*_)N (Ni) and Li_2_(Li_1−*x*_Cu_*x*_)N (Cu) single crystals was achieved through a Li-rich solution, concentrations of each transition metal ion were deduced to be *x* = 0.123(1), 0.156(2), 0.092(1), 0.167(2), 0.094(2) respectively. Detailed information on crystal synthesis is reported elsewhere.^19^ Metal-ion substitution within the α-Li_3_N matrix replaces a Li-ion situated at the 1b Wyckoff position. Li_2_(Li_1−*x*_TM_*x*_)N crystallises as a hexagonal lattice of Li_2_N layers alternating with Li_1−*x*_TM_*x*_ planes perpendicular to the crystallographic *c* axis.

### 2.2 Experimental

L_2,3_-edge XAS and XMCD measurements were performed at the I10-BLADE high-field magnet end station at Diamond Light Source, UK. Continuously scanning monochromator energy scans were acquired for each transition metal ion at the respective metal L_2,3_-edges: Mn (620–690 eV), Fe (690–755 eV), Co (750–840 eV), Ni (835–920 eV) and Cu (920–1000 eV), each at 0.1 eV energy intervals. Measurements were performed at 21 K within an ultra-high vacuum (10^−10^ bar). Total fluorescence yield (TFY) was acquired in a back-scattering geometry using a 10 × 10 mm^2^ silicon diode with a 150 nm Al cover to filter out emitted electrons. The Li_2_(Li_1−*x*_TM_*x*_)N single crystals are air sensitive. Crucibles were opened and crystals were mounted with Torr Seal to sample holders within an argon atmosphere glovebox (<0.5 ppm O_2_ and H_2_O). The samples were rapidly transferred into the XMCD load lock through a nitrogen-purged glove bag. XMCD measurements were performed at 14 T and collected through the individual detection of right (*σ*_r_) and left (*σ*_l_) circular polarisation with TFY detection. XAS measurements were acquired through linear horizontal polarisation (*σ*_h_) and TFY detection. Background subtraction of XAS spectra was performed with a linear fitting of the pre-edge and normalised through a linear fitting of the post-edge. The 2p_3/2_ and 2p_1/2_ continuum transitions were subtracted through a double arctangent function (further details of background subtraction see ESI† of Huzan *et al.*^5^).

## 3 Computational

### 3.1 *Ab initio* calculations

**Fig. 2:**
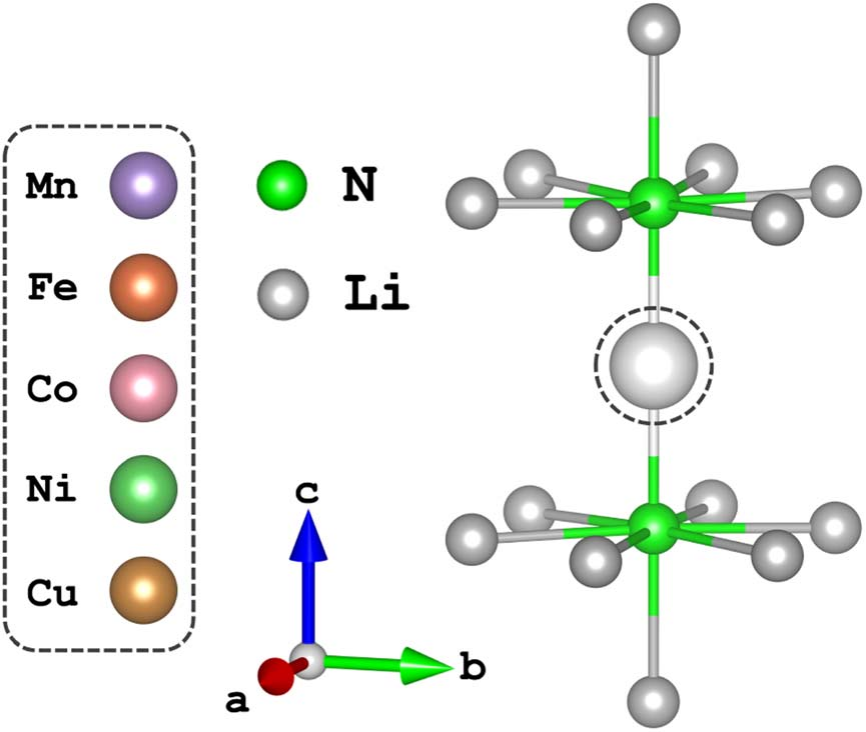
The local dopant environment, as a cation fragment [Li_14_TMN_2_]^9+^, utilised for TD-DFT and SA-CASSCF/NEVPT2 calculations where TM ions replace the Li-ion at the 1b-Wyckoff position.

Time-dependent density functional theory (TD-DFT) and complete active space self-consistent field (CASSCF)^20,21^ calculations presented in this work were performed using the quantum chemistry software suite, ORCA, version 5.0.2.^22^ Structural optimisation of N–TM–N bond lengths were calculated through energy minimisation of DFT-SCF calculations for TM = Mn, Ni and Cu of the [Li_14_TMN_2_]^9+^ fragment, Fig. 2; EXAFS deduced bond lengths were used for Fe^5^ and Co.^23^ Calculations used a combination of the def2-TZVPP, def2-TZVP and def2-SVP all-electron basis sets^24^ for the transition metal, nitrogen and lithium atoms, respectively. Scalar relativistic effects are included through the second-order Douglas–Kroll–Hess (DKH2)^25,26^ method. TD-DFT calculations applying the B3LYP^27^ functional were performed with spin multiplicity of the Cu metal centre defined as 2*S* + 1 = 1 and fragment charge of +9, Fig. 2. The molecular fragment (Fig. 2) was selected to maintain the *D*_6h_ symmetry of α-Li_3_N. There was no significant deviation in the simulated spectroscopic line shape upon varying the Cu–N bond lengths within a reasonable range. Saturation of the spectroscopic features required 150 roots per multiplet, and an empirical shift of 9.33 eV was applied to match the experimental data. State averaged (SA) CASSCF calculations in conjunction with N-electron-valence perturbation theory (NEVPT2)^28–30^ were performed with an active space of N electrons in five orbitals (*N*,5), where *N* = 6 through to 9 for TM = Mn (5 quintets, 45 triplets and 50 singlets), Fe (10 quartets and 40 doublets), Co (10 triplets and 15 singlets) and Ni (5 doublets). The SA-CASSCF/NEVPT2 results were projected using the *ab initio* ligand-field theory (AILFT)^31,32^ method available within ORCA upon the d-orbital basis set giving ligand-field parameters (D*s*, D*t*), Slater integrals (B and C) and spin–orbit coupling strengths (*ζ*_3d^*n*^_, *ζ*_2p_) for all the metal ions. These parameters were imported into Quanty to calculate the fluorescence-XAS spectra. Further details are found in the ESI.† Complementary periodic DFT computational details and L_3_-edge calculations are provided within the ESI.† SA-CASSCF/NEVPT2 calculations based on a larger six orbital active space (*N*,6) were conducted to quantify trends in Li_2_(Li_1−*x*_TM_*x*_)N d–s mixing. The active space was constructed through rotation of the unoccupied sσ orbital of the previously converged five orbital CASSCF active space to construct CASSCF(*N*,6), where *N* = 6 through to 10 for TM = Mn (1 septet, 35 quintets, 189 triplets and 175 singlets), Fe (6 sextets, 84 quartets and 210 doublets), Co (15 quintets, 105 triplets and 105 singlets), Ni (20 quartets and 70 doublets) and Cu (15 triplets and 21 singlets).

### 3.2 Multiplet calculations

Multiplet calculations were undertaken using the many-body scripting language, Quanty^33–35^ for the simulation of L_2,3_-edge fluorescence-XAS and XMCD. Slater–Condon–Shortley integrals *F*^k^_dd_, *F*^k^_pd_ (Coulomb) and *G*^k^_pd_, *G*^k^_ds_, *G*^k^_ps_ (exchange) were obtained from *ab initio* Hartree–Fock calculations and scaled down to 80% to account for the overestimated electron–electron repulsions observed within these calculations for free ions.^36–38^ Spin–orbit coupling constants (*ζ*_3d_) were taken as their atomic values where experimental data is not available. The 2p^5^ core-hole spin–orbit coupling constants (*ζ*_2p_) are consistent with the atomic values. The strength of ligand-field splitting in the *D*_∞h_ point group, 3d–4s mixing, and metal-to-ligand charge-transfer were parameterised to match the single crystal XAS and XMCD data using the result of AILFT calculations as a starting point before refinement to improve the fit with respect to the single crystal L_2,3_-edge XAS and XMCD experimental data. A complete description of the computational details is provided within the ESI.†

## 4 Results and analysis

### 4.1 L_2,3_-edge XAS, 3d–4s mixing and covalency

#### 4.1.1 Cu L_2,3_-edge XAS and DFT

**Fig. 3:**
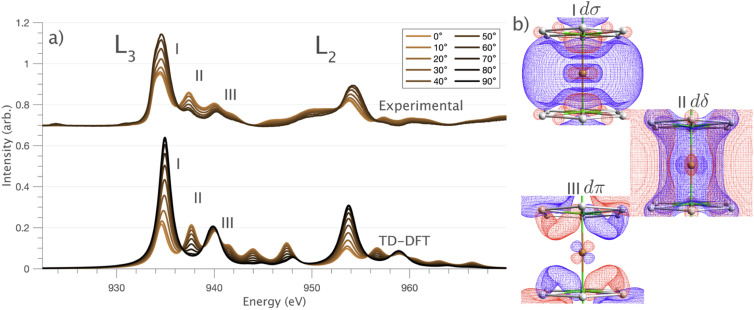
(a) Single crystal angular dependent Li_2_(Li_1−*x*_Cu_*x*_)N L_2,3_-edge XAS measured at 21 K. (Top) Experimental spectra with background subtraction. (Bottom) TD-DFT calculated spectra. 0° corresponds with 
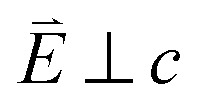
 and 90° with 
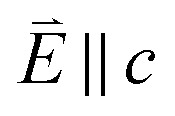
. (b) Isosurface plots of the natural transition orbitals.

Single-crystal L_2,3_-edge XMCD measurements of Cu show no dichroism at 21 K and 14 T (Fig. S1†). The absence of XMCD is consistent with a monovalent oxidation state (closed shell Cu-3d^10^). This confirms that previously reported evidence of Li_2_(Li_1−*x*_Cu_*x*_)N magnetism *via* magnetic susceptibility is due to the presence of paramagnetic impurities.^39,40^ Linearly horizontal polarised L_2,3_-edge XAS of Cu display pronounced single-crystal angular dependencies. Fig. 3a shows single-crystal Cu measurements acquired over a range of angles from 0 to 70°, where 0° corresponds with the 
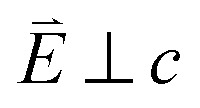
 and 90° with 
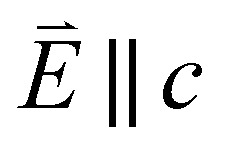
. The L_2,3_-edge involves an electric dipole allowed 2p core-electron excitation. The 2p^5^ core configuration undergoes spin–orbit coupling causing the division of the edge into the 2p_3/2_ (L_3_) and 2p_1/2_ (L_2_), which in the case of Cu is split by ∼20 eV. The L_2_-edge is ∼1.5 times broader than the L_3_-edge due to additional Coster–Kronig Auger decay channels for this excitation. Generally, transition metal L_2,3_-edge XAS is dominated by 2p → 3d dipole transitions.^41^ For Cu(i), with a fully occupied 3d shell, the L_2,3_-edge XAS exhibits weak 2p → 4s dipole transitions,^42^ and in the absence of 3d–4s hybridisation, no significant angular dependence in these transitions is expected. The L_2,3_-edge XAS of Cu, exhibits multiple transitions with strong angular dependence. The L_2_-edge (944–966 eV) and L_3_-edge (930–944 eV) are very similar; however, since the L_3_-edge has better spectral energy resolution, analysis of transitions is focused on this region of the spectra. The Cu L_3_-edge features are dominated by three (I–III) sets of peaks centred at 934.4, 937.4 and 940.1 eV, respectively. Interestingly, peaks I and II exhibit opposing angular dependence, indicating that they originate from final states with differing symmetries. The angular dependence of peak III is composed of multiple overlapping features following a less pronounced angular dependence than I and II.

DFT calculations are applied to simulate the measured spectra to assist the transition assignment and further investigate the electronic structure. Since Cu is a dopant within an extended solid, it is necessary to identify if the L_3_-edge XAS final states are localised to the immediate Cu coordination environment or if significant transition intensity relates to the band structure of the Li_3_N crystal. To address this question, molecular and periodic DFT calculations are applied to simulate the measured spectra (see ESI† for the periodic DFT calculations). Both methods are found to accurately reproduce the L_3_-edge XAS spectral shape and angular dependence (Fig. 3a and S2a†) inferring that the theoretical [Li_14_TMN_2_]^9+^ molecular fragment suitably captures the essential electronic structure of Li_2_(Li_1−*x*_TM_*x*_)N at the dopant sites. Both periodic and molecular DFT calculations aid in the assignment of the Cu spectra providing evidence of the admixture of Cu 3d character into the lowest energy unoccupied states at the dopant. Peak I has maximum intensity with the incident X-ray wave vector, 
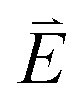
 parallel to the principle *c*-axis, and can therefore be assigned as relating to states with strong Cu 3dσ mixing. Peak II, with opposing angular dependence to I, is assigned as relating to states with Cu 3dδ mixing. Peak III has similar but less resolved angular dependencies than peak II and originates from Cu 3dπ mixing. Identification of the natural transition orbitals supports this interpretation for peaks I to III from which characteristic Cu 3d_*z*^2^_ (dσ), Cu 3d_*x*^2^−*y*^2^_,_*xy*_ (dδ) and Cu 3d_*xz*,*yz*_ (dπ) orbitals can be identified, Fig. 3b. Similarly, the partial density of states extracted from periodic DFT calculations provides further support for the assignment of peaks I to III (Fig. S2b†). An analysis of [Li_14_CuN_2_]^9+^ molecular orbitals are shown in Fig. S3† along with Löwdin population analysis (Table S1†). Unoccupied virtual orbitals with significant Cu character are similar in shape and phase relative to the natural transition orbitals for transitions I, II and III. At lowest energy there is an unoccupied σ molecular orbital composed of Cu 3d_*z*^2^_, Cu 4s, N 2p_*z*_ and N 2s character. At higher energy there is an δ molecular orbital composed of Cu 3d_*x*^2^−*y*^2^_,_*xy*_ character mixed with Li 2p_*x*,*y*_ and Li 2s character. At highest energy, there is a π molecular orbital composed of N 2p_*x*_ and 2p_*y*_ character, mixed with Cu 3d_*xz*,*yz*_ and Li 2s and 2p character. Analysis of the highest occupied molecular orbitals identify the presence of significant π bonding interactions between Cu 3d_*xz*,*yz*_ and N 2p_*x*,*y*_, with 27.2% N character mixing into the 3d_π_ bonding orbitals. Similarly, significant σ bonding interactions are identified, with 15% N 2p_*z*_ character and 15.2% Cu 4s character mixing into the 3dσ bonding orbital. Cu 3d_*x*^2^−*y*^2^_,_*xy*_ is non-bonding. This indicates that the L_2,3_-edge XAS accessed virtual δ orbital (natural transition orbital II) does not relate to the bonding character of Cu.

#### 4.1.2 Spectral trends for linear coordinated transition metal L_2,3_-edge XAS

**Fig. 4:**
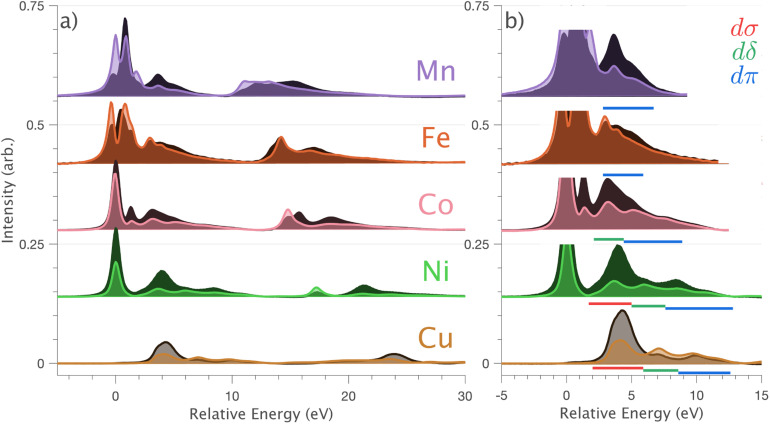
(a) Single crystal L_2,3_-edge XAS of Li_2_(Li_1−*x*_TM_*x*_)N, where TM = Mn, Fe, Co, Ni and Cu measured at 21 K. Normal incidence 
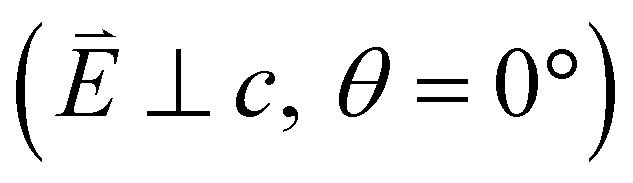
 spectra are shown in light colours and grazing incidence (*θ* = 70°) spectra are shown in dark colours. (b) The high energy side of the L_3_-edge region of the spectra is shown to emphasise charge-transfer satellites, labelled with an underline in red (dσ), green (dδ) and blue (dπ).

An overview of angular-dependent single-crystal L_2,3_-edge XAS for the Li_2_(Li_1−*x*_TM_*x*_)N series is plotted together on a relative energy scale for comparative purposes in Fig. 4. The spectra gain complexity from Cu through to Mn due to the increasing number of 3d-holes that introduce additional dipole transitions into unoccupied ligand-field multiplet final-states. The splitting of the L_2_- and L_3_-edges reduces from Cu through to Mn due to decreased transition metal 2p^5^ spin–orbit coupling strengths. The spectra of Ni, Co, Fe and Mn all exhibit a series of intense satellite features on the high energy side of the L_2_- and L_3_-edges that resemble the Cu spectrum. Fig. 4b focuses on these satellite features at the L_3_-edge region. Using the similarity in the shape and intensity angular dependence of the satellites, in going from Cu through to Mn, the dσ, dδ and dπ charge-transfer assignment for Cu can be approximately tracked down the series. For Ni, dσ, dδ and dπ are all resolved clearly and strongly resemble the Cu spectra (individual transition assignments, underlined red (dσ), green (dδ) and blue (dπ), Fig. 4b). However, for Co, Fe and Mn the satellites become increasingly masked due to overlap with more intense 2p → 3d multiplet features.

In going from Cu to Ni, large 2p → 3d dipole transitions are observed at the L_2_ and L_3_-edges, Fig. 4, consistent with a Ni(i) (3d^9^) oxidation state. The intense Ni L_3_-edge (0 eV relative energy) and less intense L_2_-edge (17.2 eV relative energy) XAS peaks have an opposing angular dependence; the L_3_-edge shows a maximum intensity with 
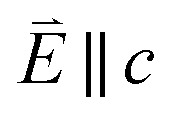
 while the L_2_-edge shows a maximum with 
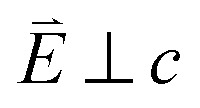
. This angular dependence is unique to the excitation relating to a final state with 3d_*xz*,*yz*_ character (see Fig. S4† which demonstrates this with Ni(i) multiplet simulations for a series of ligand-field symmetries).

Intense Co L_2,3_-edge 2p → 3d dipole transitions (0 and 17.2 eV relative energies) exhibit the same angular dependencies as Ni. This assigns the lowest unoccupied molecular orbital 3d_*xz*,*yz*_ in character. The main L_2_- and L_3_-edge peaks for Co are more intense relative to Ni, which is consistent with an additional 3d_*xz*,*yz*_-hole in going from monovalent Ni (3d^9^) to monovalent Co (3d^8^). The Co spectra also exhibit an additional peak (1.5 eV relative energy, Fig. 4) that is assigned as a 2p–3d multiplet effect present in the absorption final state in the following sections. The L_2,3_-edge XAS of Fe and Mn are more complex and require a multiplet theory interpretation, also presented in the following sections. However, despite the increasing dominance of 2p → 3d dipole transitions in going from Ni to Mn, all the spectra show strong satellite intensities at energies extending above the L_2_- and L_3_-edges resembling the Cu spectrum, Fig. 4b.

#### 4.1.3 *Ab initio* calculations

**Fig. 5:**
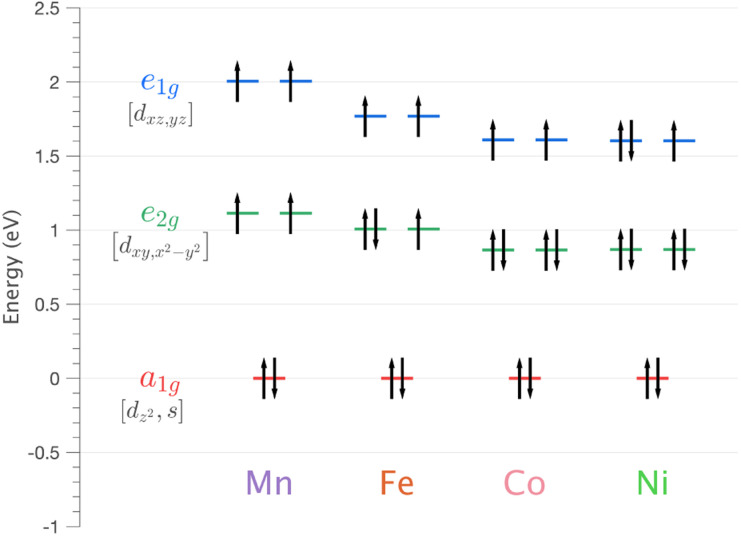
Energy of 3d orbitals for Li_2_(Li_1−*x*_TM_*x*_)N (where TM = Mn, Fe, Co and Ni) obtained *via* SA-CASSCF/NEVPT2 AILFT calculations.

CASSCF calculations based on a five-orbital active space are conducted as an aid for interpreting electronic structure trends across transition metal series. *Ab initio* ligand-field theory (AILFT) analysis is applied to obtain insight into the ligand-field, multiplet and spin–orbit coupling contributions to the electronic structure. The calculated ligand-field splitting is shown for Mn through to Ni in Fig. 5. The series exhibits a fully occupied, energy-stabilised 3d_*z*^2^_ orbital (A_1g_), degenerate 3d_*xz*,*yz*_ orbitals (E_1g_) and 3d_*xy*_,_*x*^2^−*y*^2^_ (E_2g_) orbitals. For all transition metals in the series, the calculated ligand-field splitting is large relative to other linear transition metals,^1,4,43^ gradually decreasing from Mn to Ni. The AILFT results are validated against experimental measurement *via* their implementation in ligand-field multiplet L_2,3_-edge XAS simulations. The *ab initio* ligand-field multiplet simulations of the L_2,3_-edge XAS spectra are shown in Fig. S5.† Simulation of L_2,3_-edge XAS requires a theoretical description of both initial (3d^*N*^) and absorption final state (2p^5^3d^*N*+1^) configurations. AILFT calculations, including both ground (3d^*N*^, 1-shell) and excited (2p^5^3d^*N*+1^, 2-shell) configurations were found to overestimate 2p–3d coulombic and exchange Slater integrals, resulting in a disproportionate spread of the calculated L_2,3_-edge XAS multiplet character as compared to the experimental measurements, Fig. S5d.† However, simulations utilising AILFT for the 3d-shell only in conjunction with Hartree–Fock theory-derived 2p–3d Slater integrals show good agreement with experimental results, Fig. S5b.† To confirm a monovalent oxidation state of the transition metal series, equivalent AILFT L_2,3_-edge XAS simulations are also shown, assuming a divalent oxidation state, Fig. S5c and S6.† The monovalent AILFT L_2,3_-edge XAS simulations show much better agreement with the experimental data than the divalent simulations, confirming the monovalent oxidation state for the series. The general L_2,3_-edge 2p → 3d dipole transitions are reproduced *via* AILFT. However, the simulations do not reproduce the observed satellite intensities present at the high energy side of the L_2_- and L_3_-edges. Since these satellites are assigned in Section 4.1.2 to include 3d mixing enhanced 4s excitations, a series of six orbital active space *ab initio* calculations are conducted to obtain an overview of the 4s contributions to electronic structure across the series. The amount of occupied 4s character mixed into the ground state wave-function for Mn through to Cu is shown in Table 1. The largest amount of mixing is identified for Cu and Ni at ∼23%. To further identify the sensitivity of L_2,3_-edge XAS to 3d_*z*^2^_–4s mixing and metal–ligand covalency, charge-transfer multiplet simulation is presented in the following section.

**Table 1:** Six orbital active space CASSCF ground-state wavefunction (*ψ*_*i*_) decomposition with respect to 3d^*n*^ and 4s for the Li_2_(Li_1−*x*_TM_*x*_)N series, where *n* = 6 for Mn through to 10 for Cu; all values in %

*ψ*_i_	Mn	Fe	Co	Ni	Cu
|3d^*n*^〉	86.46	84.34	82.51	76.05	75.72
|3d^*n*−1^4s^1^〉	8.43	9.54	12.07	19.74	21.87
|3d^*n*−2^4s^2^〉	4.97	5.19	3.62	3.53	2.04

#### 4.1.4 Charge-transfer ligand-field multiple calculations

**Fig. 6:**
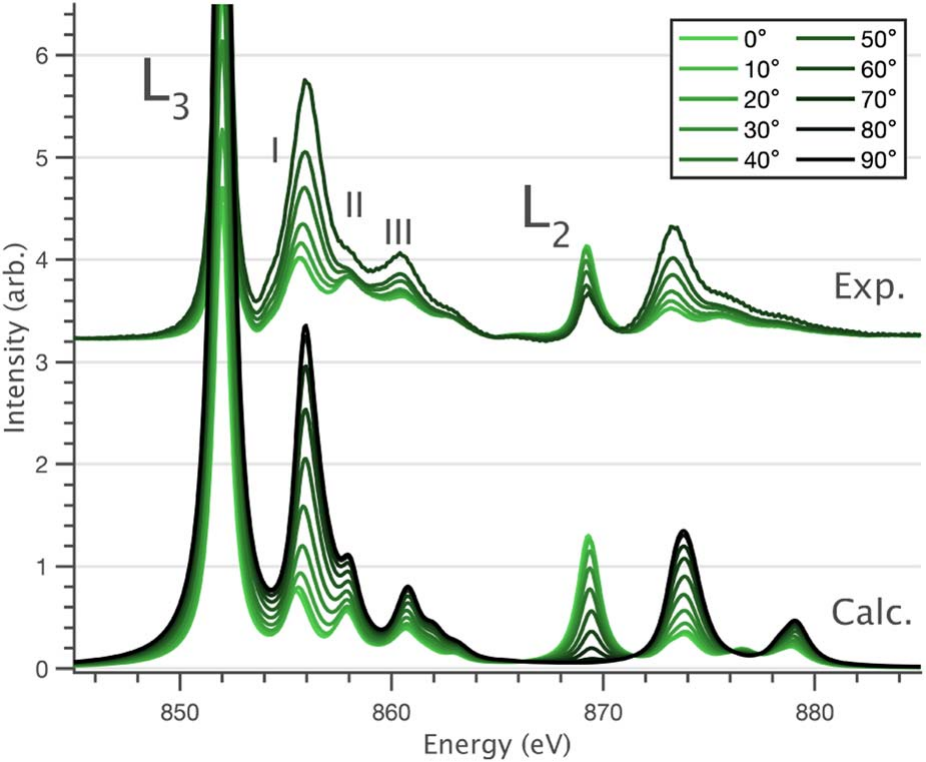
Single crystal angular dependent Ni L_2,3_-edge XAS measurements. (Top) Experimental spectra with background subtraction. (Bottom) Charge-transfer ligand-field multiplet calculations. 0° corresponds with 
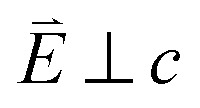
 and 90° with 
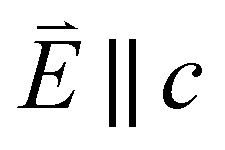
 measured at 21 K.

**Fig. 7:**
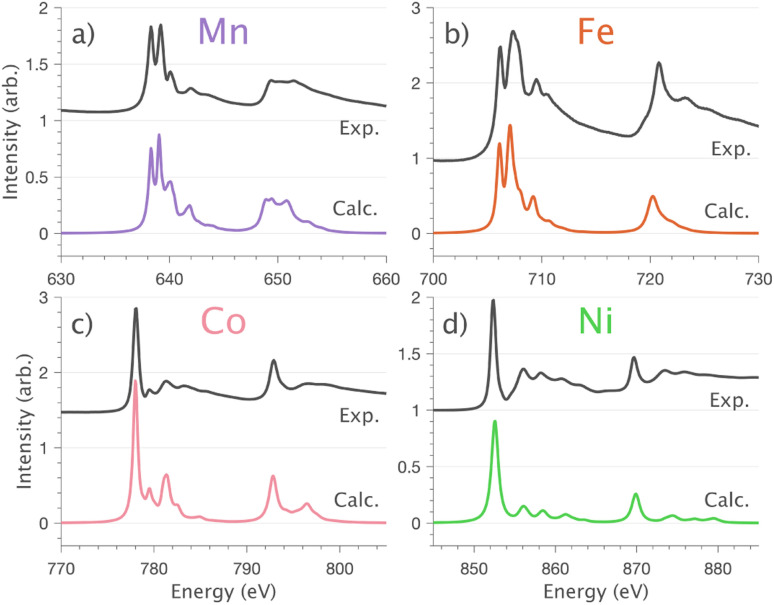
Normal incidence 
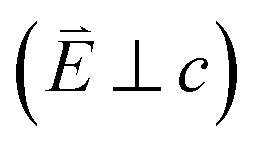
 single crystal L_2,3_-edge XAS measurements and calculations for Li_2_(Li_1−*x*_TM_*x*_)N (where TM = (a) Mn, (b) Fe, (c) Co and (d) Ni). Experimental spectra measured at 21 K (black) and optimised charge-transfer multiplet calculations (colour).

Angular dependent Ni L_2,3_-edge XAS results are shown in Fig. 6. The Ni L_2_ (871–880 eV) and L_3_-edge (853.5–867 eV) satellite intensities are very similar and show similar angular dependence sensitivities. However, since the L_3_-edge satellites are better resolved, analysis is focused on the L_3_-edge part of the spectrum. The satellites include three features, labelled I, II and III, with distinct angular dependencies (3dσ, 3dδ and 3dπ) consistent with the Cu L_2,3_-edge XAS, Fig. 3 and 6. Starting with the five orbital active space AILFT multiplet model from Section 4.1.3, valence bond configuration interactions (VBCI) are added such that the influence of Ni 3d–4s mixing and metal–ligand covalency is included, parameterisation of the VBCI model is conducted to reproduce the satellite region of the L_2,3_-edge. It is identified that 3d–4s mixing has a significant influence on the spectrum shape in the satellite region of the spectrum (see Fig. S7a†). It is also identified that the L_2,3_-edge has a sensitivity to symmetry-restricted metal–ligand orbital mixing^44^*via* nitride dπ and lithium dδ symmetry is shown in Fig. S7b and c.† The charge-transfer multiplet simulations, including 3d–4s mixing and dπ and dδ metal–ligand interactions accurately reproduce the angular dependent Ni L_2,3_-edge XAS spectra, including satellites, Fig. 6. Applying the same method to Co, Fe and Mn L_2,3_-edge XAS spectra enables accurate simulation of both absorption edges and satellites (Fig. 7), including angular dependencies (Fig. S8†).

### 4.2 L_2,3_-edge XMCD, spin–orbit coupling and magnetism

**Fig. 8:**
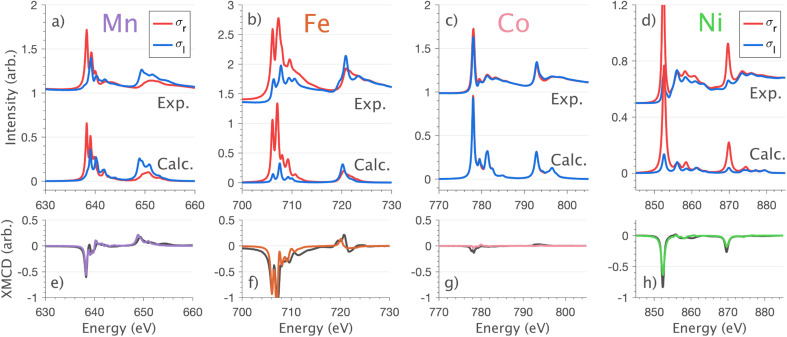
Normal incidence 
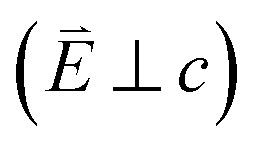
 single crystal L_2,3_-edge XMCD measurements and calculations of Li_2_(Li_1−*x*_TM_*x*_)N (where TM = Mn, Fe, Co and Ni). (a–d) Circular polarisation absorption spectra *σ*_r_ (red) and *σ*_l_ (blue) with resultant (e–h) XMCD (*σ*_r_ − *σ*_l_) spectra; experimental (black) and charge-transfer multiplet calculations (colour) measured at 21 K and 14 T.

XMCD measurements are conducted to obtain insight into spin and orbital contributions to magnetism. Normal (*H*‖*c*) and grazing (*H*⊥*c*) incidence L_2,3_-edge XMCD measurements in an applied magnetic field of 14 T show a variation in magnetic anisotropy for the transition metal series (Fig. 8 and S8†). The XMCD signal for Mn shows only weak orientation dependence. Large easy-axis anisotropy is observed for Fe and Ni, with a large XMCD signal for *H* parallel to *c* and a weak XMCD signal for *H* at grazing incidence. Easy-plane type magnetic anisotropy is observed for Co, with a small XMCD signal when *H* is applied parallel to *c* and a large XMCD signal with *H* at grazing incidence. These observations are consistent with single-crystal magnetometry measurements.^45^

Analysing the shape of the XMCD spectra provides insight into the origin of the observed magnetic anisotropy, particularly the relative sign and shape of the L_2_-edge XMCD *versus* L_3_-edge XMCD. For Ni the XMCD at both the L_2_- and L_3_-edges have the equivalent sign, Fig. 8h. A series of Ni L_2,3_-edge multiplet simulations show that the XMCD is very sensitive to the angular momentum of the ground state electron configuration, Fig. S4.† From this, it is identified that an XMCD signal with equivalent signs at both L_2_- and L_3_-edges is a characteristic fingerprint for a strongly spin–orbit coupled *S* = 1/2, *L* = 1 and *J* = 3/2 (^2^P_3/2_) ground term in Ni(i).

It is interesting that despite the Ni and Co L_2,3_-edge XAS spectra having similar shapes (Section 4.1) the XMCD differ significantly, Fig. 8g and h. Unlike Ni, the L_2_-edge XMCD of Co is positive, the opposite of the Ni L_2_-edge XMCD, indicating a partial quenching of orbital angular momentum for Co. The XMCD spectra of Fe and Mn exhibit complex structure due to ligand-field multiplet effects. Implementation of the charge-transfer ligand-field multiplet model developed in Section 4.1.4 is found to accurately reproduce the XMCD spectra of the transition metal series, including angular dependence in XMCD and the extension of the XMCD into the charge-transfer satellite region of the L_2,3_-edge, Fig. 8 and S8.† The large XMCD signal and easy-axis anisotropy for Fe are consistent with a ^4^D_7/2_ ground term as previously assigned by variable temperature L_2,3_-edge XAS.^5^

The application of sum-rule analysis through XMCD measurements is frequently utilised to decompose spin and orbital contributions to the total magnetic moment.^46,47^ However, since single-crystals of Li_2_(Li_1−*x*_TM_*x*_)N are electrically insulating, total-electron yield detection is inhibited. Therefore, the experimental results are fluorescence yield detected. Fluorescence yield exhibits state and angular-dependent fluorescence decay channels that deviate significantly from the absorption cross-section; thus limiting the utility of sum rule analysis.^48^ Instead, the charge-transfer ligand-field multiplet results are analysed to obtain the lowest energy expectation values for *m*_*S*_, *m*_*L*_ and *m*_*J*_ for each of the compounds. Table 2 presents the energy and related *m*_*S*_, *m*_*L*_ and *m*_*J*_ expectation values for Mn through to Ni. The weak magnetic anisotropy for Mn and easy-plane anisotropy for Co can be understood due to their orbital moment quenched ground states, resulting from a^2^_1g_e^2^_2g_e^2^_1g_ and a^2^_1g_e^4^_2g_e^2^_1g_ configurations, respectively. A positive axial zero-field splitting of the *S* = 2 and *S* = 1 ground-state multiplets for Mn and Co, respectively, are responsible for the easy-plane type magnetisation observed. The more substantial easy-plane anisotropy for Co *versus* Mn can be explained by the more significant zero-field splitting present (see Zeeman diagrams, Fig. S9†).

**Table 2:** Eigenvalues and expectation values for the lowest-energy states of relevance to single-ion anisotropy, as shown in Fig. 9 for Li_2_(Li_1−*x*_TM_*x*_)N. *E*, *m*_*S*_, *m*_*L*_, and *m*_*J*_ correspond to expectation values, energy (eV), spin, orbital angular momentum and total angular momentum

TM	〈*E*〉	〈*m*_*S*_〉	〈*m*_*L*_〉	〈*m*_*J*_〉
Mn	0.00	0.000	0.000	
	0.25	±1.022	±0.002	
	0.98	±2.045	±0.002	
Fe	0.00	±1.505	±2.002	±3.506
	31.88	±0.504	±2.001	±2.505
	65.92	±0.498	∓1.999	∓1.501
	101.67	±1.503	∓2.000	∓0.497
Co	0.00	0.000	0.000	
	4.14	±0.910	±0.010	
Ni	0.00	±0.486	±0.948	±1.471
	75.66	±0.501	∓0.933	±0.432

**Fig. 9:**
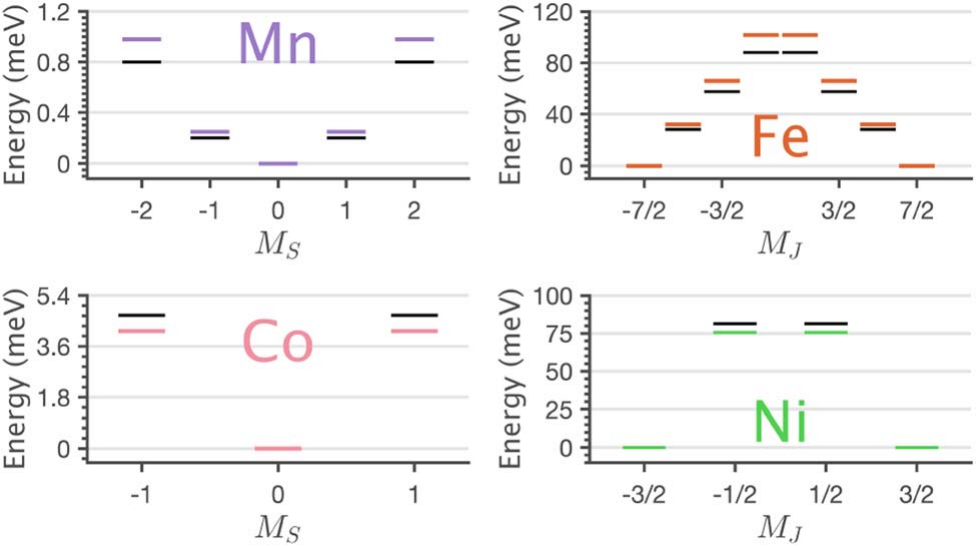
Charge-transfer ligand-field multiplet (colour) and CASSCF-QDPT-SOC. (black) Determined energy level diagrams of Li_2_(Li_1−*x*_TM_*x*_)N exhibiting easy-planar, Mn and Co, and easy-axis, Fe and Ni anisotropy barriers to magnetisation reversal.

The easy-axis magnetic anisotropy of Fe and Ni derives from an odd electron count in doubly degenerate orbitals. An a^2^_1g_e^3^_2g_e^2^_1g_ configuration for Fe and an a^2^_1g_e^4^_2g_e^1^_1g_ configuration for Ni. The ^4^D_7/2_ ground term for Li_2_(Li_1−*x*_Fe_*x*_)N splits in energy into sub-states *m*_*J*_ = ±7/2, ±5/2, ±3/2, ±1/2 resulting in a large energy barrier to magnetisation reversal, Table 2. Interestingly, the Ni ^2^P_3/2_ ground term is calculated to have an extremely large barrier to magnetisation reversal, generated by the splitting of *m*_*J*_ = ±3/2, ±1/2 sub-states, resulting in a predicted energy barrier of *U*_eff_ ≈ 75 meV (≈600 cm^−1^). To our knowledge, this exceeds the energy barrier to magnetisation reversal for any reported transition metal single-ion. Fig. 9 shows the lowest energy eigenstates obtained from charge-transfer multiplet simulations along with *ab initio* energies extracted from the 5 orbital active space CASSCF *via* quasi-degenerate perturbation theory (QDPT) with spin–orbit coupling. Good agreement is obtained between the semi-empirical and *ab initio* results for the transition metal series.

## 5 Discussion

L_2,3_-edge XAS 2p → 3d dipole transitions are sensitive to 3d ligand-field splitting, multiplet effects, and the presence of metal–ligand covalency. Metal–ligand covalency gives rise to charge-transfer satellites on the high energy side of the absorption edges that are considered hallmarks for ligand donor and back donor covalency.^49^ The dipole 2p → 4s contribution to the L_2,3_-edge XAS is generally neglected since it carries much less intensity and is much broader than the 2p → 3d transitions.^42^ This study shows that in a linear coordination environment, significant mixing of 3d_*z*^2^_ character into 4s occurs. This gives rise to 3d_*z*^2^_ intensity enhanced 2p → 4s transitions observed as satellites on the high energy side of the L_3_ and L_2_-edges. The 3dσ enhanced 4s satellites are most evident in the L_2,3_-edges of Cu and Ni, which is consistent with *ab initio* calculations that identify maximum 4s mixing in ground state wave functions for Cu and Ni (∼23% occupied 4s character), with significantly less 4s mixed into the ground states for Co (∼16%), Fe (∼15%) and Mn (∼13%). For Fe and Mn, intense 2p → 3d multiplet features distribute over a large energy range masking direct assignment of 3d_*z*^2^_–4s mixing-induced satellites. However, in principle, this intensity overlap issue could be ameliorated by resonant inelastic X-ray scattering (RIXS). Indeed given the broad interest in d–s mixing, it will be interesting to identify if RIXS can be applied to quantify d–s mixing in other scenarios, including transition metals,^50^ f-block single-ion magnets,^14,15^ qubits and actinides^12,16^ predicted to exhibit f^*n*^d/s^1^ ground-state electron configurations.

Using single-crystal angular dependent measurements assists in assigning charge-transfer satellites of diffing symmetry. Metal–ligand charge-transfer satellites of 3dδ and 3dπ symmetry are also identified in addition to 3dσ satellites. Satellites of 3dπ symmetry are resolved for the whole Li_2_(Li_1−*x*_TM_*x*_)N series. Charge-transfer multiplet theory (for Mn to Ni) and DFT analysis (for Cu) show that 3dπ symmetry satellites result from covalent interactions between 3d_*xz*,*yz*_ and N 2p_*x*,*y*_ orbitals. Satellites of 3dδ symmetry are found *via* analysis of Cu DFT to involve virtual orbitals with mixed characters of 3d_*xy*,*x*^2^−*y*^2^_ and Li 2s/Li 2p_*x*,*y*_. However, the 3dδ symmetry satellites do not relate to metal–ligand covalency; according to Cu DFT results, the lower energy occupied Cu 3d_*xy*,*x*^2^−*y*^2^_ orbitals are non-bonding.

*Ab initio* ligand-field multiplet simulations of the L_2,3_-edge XAS confirm the accuracy of CASSCF calculations for predicting the ligand-field splitting. These calculations show that the total ligand-field energy splitting is ∼1.6 eV for Ni and increases along the series to ∼2.0 eV for Mn. These are exceptionally large ligand-field energy splitting for two-coordinate compounds. For instance, [Fe(C(SiMe_3_)_3_)_2_]^−^ and [Fe(N(SiMePh_2_)_2_)_2_]^−^ have total ligand-field energy splittings of only ∼0.62 eV (ref. 1) and ∼0.68 eV (ref. 51) respectively. Furthermore, a [M(N(SiMe_3_)_2_)_2_]^−^ series where M = Cr, Mn, Fe and Co show the same trend of increasing ligand-field energy splitting in going from Co to Cr; however, the total ligand-field energy splitting does not exceed 1.0 eV for any of the compounds.^52^ The local coordination of Li_2_(Li_1−*x*_TM_*x*_)N differs from these molecular complexes in that the metal–ligand bond lengths are shorter; for instance, the metal–nitrogen bond length for Fe is 1.873(7) Å (ref. 5) and Co is 1.80(1) Å,^23^ significantly shorter than 1.9213(6) Å for [Fe(N(SiMe_3_)_2_)_2_]^−^ and 1.8979(11) Å for [Co(N(SiMe_3_)_2_)_2_]^−^. The high point group symmetry and shorter metal–ligand bond lengths for Li_2_(Li_1−*x*_TM_*x*_)N, are consistent with strong dπ bonding interactions and 3d_*z*^2^_–4s mixing that drives the exceptionally large ligand-field splitting observed.

XMCD analysis provides a definitive explanation for the magnetism by deconvoluting spin and orbital contributions to magnetisation for the series (Table 2). Mn has an orbital angular momentum quenched *S* = 2 ground state, and Co has an orbital angular momentum quenched *S* = 1 ground state, with positive axial zero-field splitting responsible for observed easy-plane magnetisation. The latter agrees with previous EPR measurements.^53^ The XMCD results show the presence of first-order spin–orbit coupling for both Fe (^4^D_7/2_) and Ni (^2^P_3/2_). The identification of first-order spin–orbit coupling for Ni differs significantly relative to the linear Ni(i) molecule, [Ni(N(SiMe3)Dipp)2]^−^, which is reported to have a quenched orbital angular momentum (*S* = 1/2, *L* = 0), due to the reduced influence of 3d_*z*^2^_–4s mixing and a e^4^_2g_e^4^_1g_a^1^_1g_ ground state configuration.^54^ Strong spin–orbit coupling for Fe and Ni mean that at low doping concentrations, the dopants are single-ion magnets^5^ with significant energy barriers to magnetisation reversal (*U*_eff_). The *U*_eff_ values are not measured directly by XAS or XMCD. However, the *ab initio* calculations and multiplet simulations that reproduce the essential features of the measured XAS and XMCD spectra predict *U*_eff_ ≈ 28 meV (≈226 cm^−1^) and *U*_eff_ ≈ 75 meV (≈600 cm^−1^) for Fe and Ni respectively. Interestingly, Fe exhibits a sizeable magnetic hysteresis and the slowest magnetic relaxation of any transition metal SIM, *τ* ∼ 10^7^ s at low temperatures,^8^ whereas Ni shows no magnetic hysteresis down to 2 K.^45^ This implies that Ni exhibits significant quantum tunnelling of magnetisation within the ground state *m*_*J*_ = ±3/2 doublet while for Fe tunnelling within the *m*_*J*_ = ±7/2 ground doublet is forbidden. The XAS and XMCD measurements demonstrate that all dopants exhibit a *D*_6h_ coordination environment. Therefore, the difference in magnetic relaxation behaviour does not originate from reduced local symmetry. However, from a metal–ligand covalency and spin–orbit coupling perspective Fe and Ni differ. For Fe the ground state electron configuration has odd electron occupation in the non-bonding e_2g_ orbitals, while for Ni, the ground state electron configuration has odd electron occupation in the e_1g_ orbitals which are identified by L_2,3_-edge XAS to be engaged in significant 3dπ bonding interactions. In the case of Fe, it is proposed that the short Fe–N bond and related strong 3dπ bonding contribute to suppressing vibronic effects, resulting in increased magnetic relaxation times with respect to other linear SIMs. In the case of Ni, it is proposed that the e_1g_ 3dπ bonding interactions could enhance sensitivity to small vibronic distortions resulting in the mixture of lower symmetry character into the ground state that enhances relaxation *via* quantum tunnelling of magnetisation. Further, detailed magnetic relaxation measurements on Ni are required to investigate its magnetic relaxation dynamics in greater detail.

## 6 Conclusion

A series of transition metal dopants (Mn, Fe, Co, Ni and Cu) in lithium nitride are studied by angular dependent single-crystal L_2,3_-edge XAS and XMCD measurements combined with ligand-field multiplet and *ab initio* calculations. The dopant sites are all found to be monovalent, occupying a *D*_6h_ symmetry site with linear coordination. L_2,3_-edge XAS is developed to be a probe 3d_*z*^2^_–4s mixing *via* the quantification of ligand-field splitting and the presence of 3d_*z*^2^_ intensity enhanced 2p → 4s satellite transitions at the high energy side of both L_3_- and L_2_-edges. Analysis of XAS with support from *ab inito* calculations determines the presence of significant 3d_*z*^2^_–4s mixing across the transition metal series with maximal mixing for Cu and Ni that then decreases along the series to Mn. L_2,3_-edge XAS and DFT analysis identify strong 3dπ metal–ligand covalency across the series, whereas 3dδ orbitals are non-bonding. The presence of significant 3dπ metal–ligand covalency with substantial 3d_*z*^2^_–4s mixing is determined to be responsible for the exceptionally large ligand-field splitting quantified by XAS and *ab inito* calculations. L_2,3_-edge XMCD is applied to decompose spin and orbital contributions to easy-plane (Mn and Co) *versus* easy-axis (Fe and Ni) magnetic behaviour. The analysis confirms the origin of single-ion magnetism and slow magnetic relaxation in Fe and for Ni. First-order spin–orbit coupling resulting in such large barriers to magnetisation reversal in solid-state crystals is of relevance for the advancement of high-performance magnets free from rare-earth metals.

## Data availability

Data for this paper, including experimental data and example calculation input files are available on Figshare at https://doi.org/10.48420/24231301.

## Author contributions

MLB conceptualised and supervised the project. XAS and XMCD measurements were conducted by MLB, TGB, PB and SKC. MSH performed data analysis and conducted calculations under the supervision of MLB. MA conducted periodic DFT calculations. MF and FAB synthesised the crystals under the supervision of AJ. MSH and MLB wrote the manuscript with review and editing from all the authors.

## Conflicts of interest

There are no conflicts to declare.

## Supplementary Material

SC-015-D3SC06308A-s001
