# Supplementary material for: Quantifying the influence of 3d–4s mixing on linearly coordinated metal-ions by L2,3-edge XAS and XMCD
Source: Chem Sci. 2023 Dec 26;15(7):2433–42. doi: 10.1039/d3sc06308a (PMC10866348; doi:10.1039/d3sc06308a)
Supplement: SC-015-D3SC06308A-s001 [file SC-015-D3SC06308A-s001.pdf]

Electronic Supplementary Material (ESI) for Chemical Science.
This journal is © The Royal Society of Chemistry 2023

Electronic Supplementary Information:

Quantifying the influence of 3*d*-4*s* mixing on linearly coordinated metal-ions by $L_{2,3}$-edge XAS and XMCD

Myron S. Huzan,[a b] Timothy G. Burrow,[a b] Manuel Fix,[c] Franziska A. Breitner,[c] Sut Kei Chong,[a b] Matteo Aramini,[d] Peter Bencok,[d] Anton Jesche,[c] Michael L. Baker[a b]

[a] *Department of Chemistry, The University of Manchester, Manchester, M13 9PL, UK*

[b] *The University of Manchester at Harwell, Diamond Light Source, Harwell Campus, OX11 0DE, UK*

[c] *EP VI, Center for Electronic Correlations and Magnetism, Institute of Physics, University of Augsburg, D-86159 Augsburg, Germany*

[d] *Diamond Light Source, Harwell Science and Innovation Campus, Chilton, Didcot, OX11 0DE, UK*

Contents

S.1 Cu $L_{2,3}$-edge XMCD 3

S.2 Cu $L_{2,3}$-edge Periodic DFT 4

S.2.1 Computational Details 4

S.2.2 Cu $L_{2,3}$-edge analysis 5

S.3 Löewdin population analysis of Cu DFT 6

S.4 Ni(I) absorption cross-section selection rules 8

S.5 *Ab initio* and charge-transfer ligand-field multiplet calculations 10

S.5.1 Computational Details 10

S.5.2 *Ab initio* ligand-field multiplet calculations 12

S.6 Ni(I) charge-transfer ligand-field multiplet dependencies 13

S.7 Grazing incidence XAS and XMCD 19

S.8 Zeeman Diagrams 20

S.1 Cu $L_{2,3}$-edge XMCD

Figure S1 presents experimental $Li_2(Li_{1-x}Cu_x)N$ $L_{2,3}$-edge XMCD spectra. The absence of any dichroism is indicative of a system exhibiting no magnetic phenomena upon application of a 14 T field. This strongly suggests a near-closed shell Cu(I), d^{10} valence with spectral features corresponding to orbital and ligand hybridisation; full discussion within the main body of text.

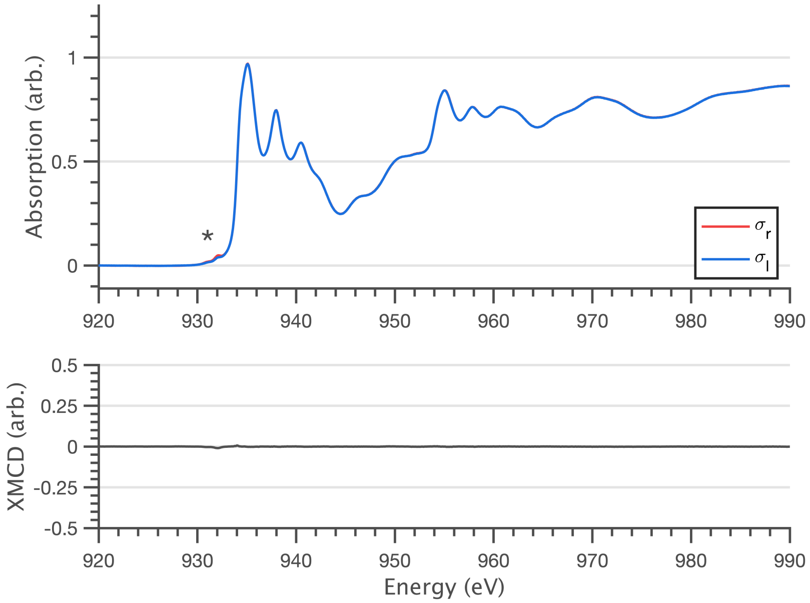

Figure S1: Single crystal $Li_2(Li_{1-x}Cu_x)N$ $L_{2,3}$-edge spectra. (*Top*) Normal incidence, $\vec{E} \perp c$, circularly polarised absorption, (σ_l and σ_r) and (*bottom*) XMCD (σ_l - σ_r) measured at 21 K and 14 T. * Small Cu(II) impurity identified within Cu(I) spectra.

S.2 Cu $L_{2,3}$-edge Periodic DFT

S.2.1 Computational Details

Periodic DFT calculations on $Li_2(Li_{1-x}Cu_x)N$ were performed using the plane-wave pseudopotential DFT method available within the code CASTEP [1]. The generalised gradient approximation for the exchange-correlation energy was selected in the form of a PBE functional revised for solids [2]. Self-consistently generated ultrasoft pseudopotentials were used for both PBE and PBE+U calculations. A kinetic energy cutoff of 750 eV for the wave function, together with a (6x6x6) Monkhorst-Pack *k*-point grid, were determined as parameters for convergence calculations. A (10x10x10) *k*-point grid was used instead for calculating the density of states (DOS). Self-consistent calculations were performed to a convergence value of 1×10^{-7} eV. Due to the isolated nature of Cu atoms in $Li_2(Li_{1-x}Cu_x)N$, we operated with a supercell constructed from the hexagonal cell of Li_3N (space group P6/mmm) with a single dopant atom. The structure was generated using the sample method as previously reported for $Li_2(Li_{1-x}Fe_x)N$ [3]. A smearing of 0.1 eV was applied to the computed eigenvalues to improve the *k*-point convergence. The angular dependence of Cu L_3-edge was calculated, including the effects of a core-hole [4] and using the same *k*-point grid as previously used for the DOS. The ground state DFT was also expanded by expressing the exchange-correlation potential in terms of local-density band theory *via* the PBE+U method [5]. The electronic properties were calculated with the simplified, rotational-invariant formulation developed within the linear response approach [6]. An effective U value of 3 eV was included in the calculations. Based on the ground-state energy evaluation and the spectroscopic results, angular-momentum dependent orbital occupation was determined with Löewdin charge analysis on top of ground-state, converged DFT wavefunctions. X-ray absorption spectra were computed by extracting the matrix elements for electronic interband transitions from the ground state DFT, including the local effects of the $2p^5$ core-hole as implemented in the CASTEP code. An energy shift of 933.38 eV was applied to match the experimental data and normalised through trapezoidal integration of the simulated spectrum. Transition broadening as a consequence of instrumental resolution (Gaussian) and core-lifetime effects (Lorentzian) was set as 0.2 and 0.7 eV FWHM, respectively.

S.2.2 Cu $L_{2,3}$-edge analysis

Periodic DFT calculations were performed within CASTEP[1] utilising a 3x3x3 supercell Li_3N matrix doped with a single Cu atom at the Wyckoff 1***b*** position. Partial density of states (pDOS) were mapped upon the converged system to compare the interpretation to molecular DFT approaches within the main body of the text. pDOS calculations permit an extension to Mulliken population analysis to isolate individual bands and orbitals of a selected atom. Unoccupied Cu-3*d* states according to molecular bonding symmetries ($d\sigma$, $d\delta$ and $d\pi$) are in agreement with both angular dependent spectroscopic labelling and molecular DFT (Figure 3) approaches to the pronounced transitions observed within the Cu L_3-edge spectra (**I-III**), Figure S2.

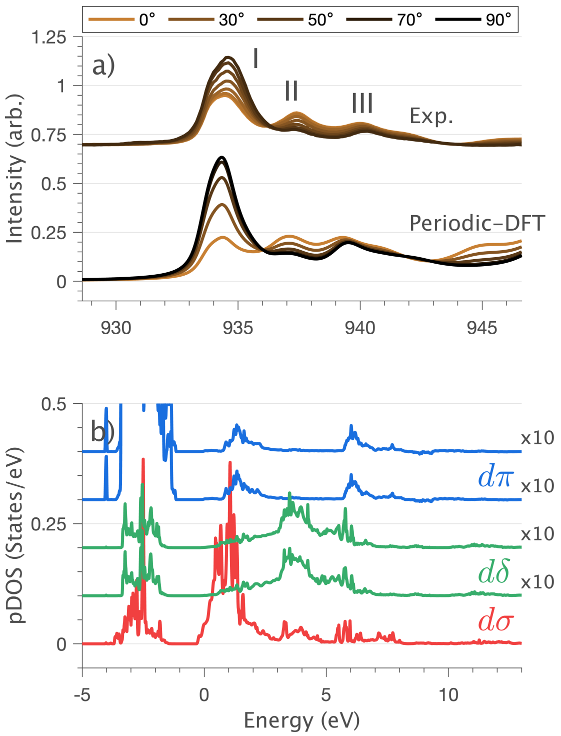

Figure S2: *a*) Single crystal angular dependent Cu L_3-edge spectra. (*Top*) Experimental spectra with background subtraction. (*Bottom*) Periodic DFT calculated spectra. 0° corresponds with **E** $\perp$ c and 90° with **E** $\parallel$ c. *b)* Periodic DFT calculated partial density of states (pDOS), visualising predominant contributions of Cu-*d* states above the Fermi energy level.

S.3 Löewdin population analysis of Cu DFT

Table S1 presents metal and ligand molecular orbital characters as calculated through Löewdin population analysis. Isosurface plots for each molecular orbital is presented within Figure S3. For conciseness, orbital character is limited to percentage character above 1%.

Table S1: Metal and ligand Löewdin deduced molecular orbital characters (%) of **Cu** DFT calculations. Isosurface plots of each molecular orbital are presented in Figure S3.

	27	28	29	30	31	34	36	37	40	41	42	51	52
Cu s	2.9					15.2			4.9				
Cu dz2	34.9					48.5			25.1				
Cu x2-y2				99.3						30.7			
Cu xy					99.3						30.7		
Cu xz		65.9						41.0				1.9	
Cu yz			65.9				41.0						1.9
N s									8.4				
N pz	51.8					15			12.4				
N px		27.2										8.2	
N py			27.2										8.2
Li s										6.8	7.2	20.8	20.8
Li pz												28.8	28.8
Li px										38.4	16.8	36.4	
Li py										16.8	36.4		36.4

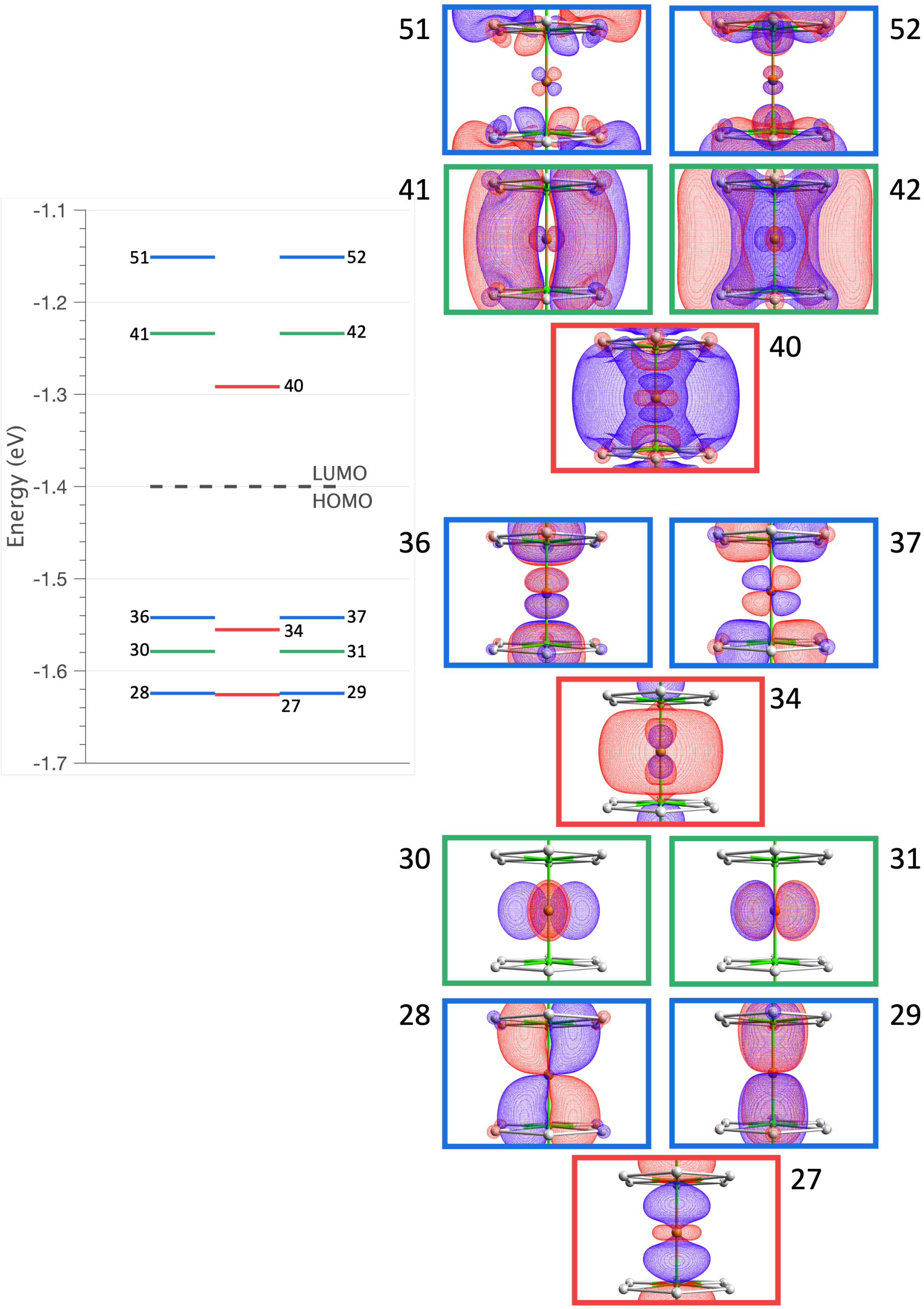

Figure S3: DFT deduced molecular orbital energy level diagram with related isosurface plots. Related Löewdin population analysis are tabulated with respect to molecular orbital number within Table S1.

S.4 Ni(I) absorption cross-section selection rules

Figure S4 presents angular dependent Ni(I) (d^9) $L_{2,3}$-edge XAS and XMCD calculations of various ground-state ligand-field configurations. The main L_3- and L_2-edge dipole transitions at 852 and 869.2 eV respectively exhibit pronounced angular dependencies which are contingent to the orbital-location of the lone electron hole. The presence of Ni(I) L_2-edge absorption intensity within the experimental spectra (Figure 8h) precludes a Ni(I) ground term $^2D_{3/2}$ ($d\sigma^2 d\pi^4 d\delta^3$) (Figure S4e); due to a lack of L_2-edge peak within the calculation. Further isolation of the orbital-location of lone electron hole can be deduced through identification of the opposing angular dependencies within the experimental measurements of the XAS L_2- and L_3-edges (Figure 6). This compels an electronic ordering of $d\sigma < d\delta < d\pi$ or $d\delta < d\sigma < d\pi$; as an unoccupied $d\sigma$ orbital ($L = 0$) predicts equivalent XAS angular dependencies (Figure S4c) in addition to an incorrect negative-positive XMCD $L_{2,3}$-edge signal, Figure S4h. This technique facilitates an understanding of the general electronic occupation of Ni(I); but it is limited to primitive labelling of the lone electron hole to an E_{1g} doublet. To further quantify the electronic ordering of the complete *d*-orbital manifolds *ab initio* and charge-transfer ligand-field multiplet calculations are applied within the main-body of the text.

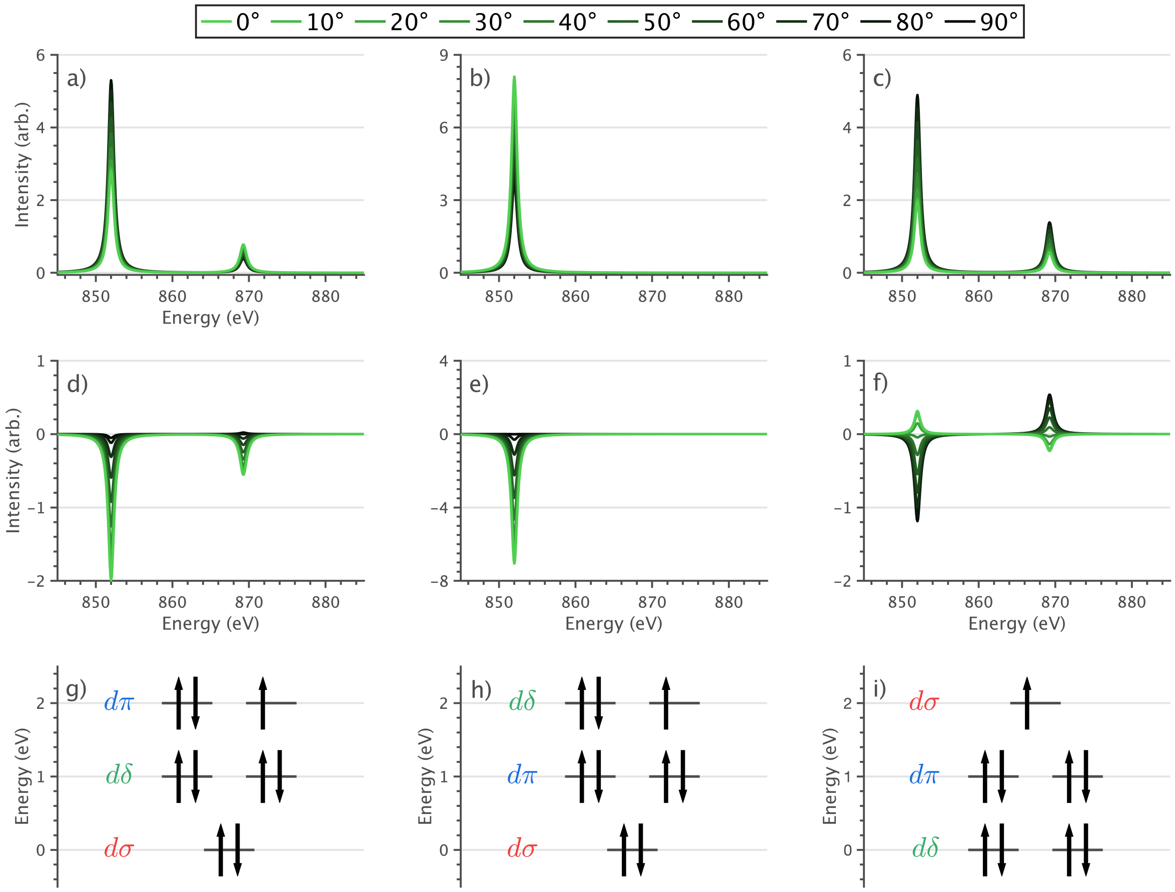

Figure S4: Ni $L_{2,3}$-edge ligand-field multiplet calculations of (a, b, c) X-ray absorption, (d, e, f) and X-ray magnetic circular dichroism calculations with (g, h, i) representative electronic (d^9) occupations. Iterative angular dependent calculations performed with incident, $\vec{E} \perp c$ (0°) through to $\vec{E} \parallel c$ (90°) at 21 K and 14 T.

S.5 *Ab initio* and charge-transfer ligand-field multiplet calculations

S.5.1 Computational Details

Charge-transfer ligand-field multiplet calculations of the $Li_2(Li_{1-x}TM_x)N$ series were performed using the quantum many-body scripting language, Quanty[7,8] to calculate $L_{2,3}$-edge XAS and XMCD spectra. Input files for the simulation of XAS and XMCD were adapted from templates generated in Crispy[9].

Quanty permits construction and solution through a Green's theorem approach of a charge-transfer ligand-field multiplet Hamiltonian as defined by:

$$\hat{\mathcal{H}} = \hat{\mathcal{H}}_{e^-e^-} + \hat{\mathcal{H}}_{SO} + \hat{\mathcal{H}}_{CF} + \hat{\mathcal{H}}_{Hyb.} \tag{S1}$$

Atomic multiplets, $\hat{\mathcal{H}}_{e^-e^-}$ are described through the expansion and separation of spherical harmonics and radial integrals into Slater-Condon-Shortley integrals, F^k_{pp}, F^k_{pd} (Coulomb) and G^k_{pd} (exchange) as given by:

$$\hat{\mathcal{H}}_{e^-e^-} = \sum_{i>j}^{N} \frac{e^2}{r_{ij}} = \left\langle {}^{2S+1}L_J \middle| \frac{e^2}{r_{ij}} \middle| {}^{2S+1}L_J \right\rangle = \sum_k f_k F^k + \sum_k g_k G^k \tag{S2}$$

where f_k and g_k represent the angular coefficients, and F^k and G^k are the radial integrals of the direct and exchange interactions, respectively. Spin-orbit coupling parameters, $\hat{\mathcal{H}}_{SO} = \sum_{i=1}^{N} \xi(r_i)\, l_i \cdot s_i$ of the $3d^n$ manifolds for Mn and Ni, are left consistent with atomic values; while Fe and Co are scaled to experimentally deduced values previously reported by temperature-dependent $L_{2,3}$-edge XAS[3] and EPR[10]. $2p^5$ core-hole spin-orbit coupling strengths of all transition metal ions remain consistent with the atomic values. The crystal-field potential, $\hat{\mathcal{H}}_{CF} = V_{CF}(r_i, \theta_i, \varphi_i) = \sum_{k=0}^{\infty} \sum_{m=-k}^{m=k} A_{k,m} r^k C^k_m(\theta, \varphi)$ within normalised spherical harmonics is solved for the local symmetry of the transition metal complex as previously defined within the $D_{\infty h}$ point group, which is equivalent to D_{6h} when $Dq = 0$. This results in a ligand-field d-orbital energy splitting of an $A_{1g}(d_{z^2})$ singlet, and two E doublets, $E_{1g}(d_{xy}, d_{yz})$ and $E_{2g}(d_{x^2-y^2}, d_{xy})$ with energies that are adjusted through the ligand-field parameters, Ds and Dt. Metal-ligand covalency is σ symmetry permitted via A_{1g}, π symmetry permitted via E_{1g} and δ symmetry permitted via E_{1g}. Orbital covalency is included within the model *via* a metal-to-ligand charge-transfer (MLCT) interaction acting between $3d^n$ and $3d^{n-1}L^-$ configurations, where L is a supplementary set of $(3d)$ ligand orbitals symmetry

permitted for metal-ligand mixing. MLCT or ligand-to-metal charge transfer (LMCT) can be used to simulate the effect of metal-ligand covalency on the measured spectra. The energy separation between configurations, Δ_L, and valence bond configuration interaction mixing is given by V_L [11] where a ground-state wavefunction, ψ_{gs} is determined through diagonalisation of the hybridisation Hamiltonian matrix:

$$\hat{\mathcal{H}}_{Hyb.} = \begin{bmatrix} 0 & V_L \\ V_L & \Delta_L \end{bmatrix}$$

$$\psi_{gs} = \alpha \left|3d^n\ \right\rangle + \beta \left|3d^{n-1}L^-\ \right\rangle$$

$$\frac{\beta}{\alpha} = \frac{\sqrt{\Delta_L^2 + 4V_L^2} - \Delta_L}{2V_L}$$

Where required, differential orbital symmetry adapted metal to ligand charge-transfer [12] is introduced to reproduce back-bonding contributions of the π and δ-bonds. Ligand back-donation is treated with parameters, V_π and V_δ and individual Δ values, Δ_π and Δ_δ. Contributions of $3d$-$4s$ hybridisation is symmetry restricted to the $d\sigma$-orbital ($3d_{z^2}$) and introduced with an energy separation, Δ_{4s} and orbital overlap parameter, V_{4s}. Additionally, further exchange Slater-Condon-Shortley integrals are included in the ground, G^2_{ds} and excited states, G^1_{ps}, G^2_{ds}. The ground state wavefunction is expressed as multiconfiguration linear combination of $\left|3d^n\right\rangle, \left|3d^{n-1}4s\right\rangle$ and $\left|3d^{n-1}L^-\right\rangle$ and was systematically explored by fitting calculated spectra to experiment, to comprehensively deduce the independent bonding contributions for the series of linear transition metal complexes studied. The charge-transfer ligand-field multiplet parameters were informed by the Cu TD-DFT calculations (discussed within the main body of text) including the relative energy ordering of characteristic satellite features of $d\sigma < d\delta < d\pi$.

Ab initio ligand-field theory calculations provide a first measure of Slater-Condon-Shortley integrals weightings from first principles, Table S3, from which optimised parameters were itteratively deduced, Table S2. Both ground-state (1-shell) and excited-state (2-shell) calculations were performed where the latter was computationally feasible. The active space of a 1-shell calculation is as described within the main body of the text, where the 2-shell enables the calculation of $2p3d$ Coulomb and exchange integrals. Rotation of the three $2p_{x,y,z}$ orbitals within the active space expands the calculation to N electrons in eight orbitals, where N = 13 and 14 for TM = Fe (56

quartets and 168 doublets) and Co (28 triplets and 36 singlets) for the respective monovalent calculation and TM = Co (56 quartets and 168 doublets) and Ni (28 triplets and 36 singlets) for divalent; tabulated values collated within Table S3. An 80% reduction is applied to Hartree-Fock deduced parameters resulting from the over-estimation of electron-electron repulsion found for the free ion where 2-shell calculations were impractical. Broadenings of all calculated transitions were convolved with a full-width half-maximum Gaussian of 0.25 eV representative of the experimental instrument resolution, and a varying Lorentzian broadening over the L_3 and L_2-edges to account for the core-hole lifetimes, Table S5.

S.5.2 *Ab initio* ligand-field multiplet calculations

Ab initio ligand-field theory calculations were performed on $Li_2(Li_{1-x}TM_x)N$ where TM = Mn, Fe, Co and Ni to gain a parameter-free insight of the series. Both mono ($[Li_{14}TMN_2]^{9+}$) and divalent ($[Li_{14}TMN_2]^{10+}$) TM fragment calculations were performed with Slater integrals, spin-orbit coupling strengths and ligand-field splitting results (Table S2) used as input values for a representative angular dependent $L_{2,3}$-edge XAS multiplet calculation, Figure S5. The SA-CASSCF-NEVPT2 AILFT calculations are most representative of the monovalent oxidation state reproducing the primary multiplet excitations (0 - ~4 eV) of the series. The higher energy satellite intensities are absent within these calculations resulting from the limited active space (5 *d*-orbitals) as described within the main body of the text, thus indicating the requirement for an extended multi-configurational multiplet Hamiltonian to comprehensively replicate the observed spectral features. Attempts at excited-state (2-shell) AILFT calculations were unsuccessful in accurately replicating the observed XAS spectra and require further exploration beyond this study's scope.

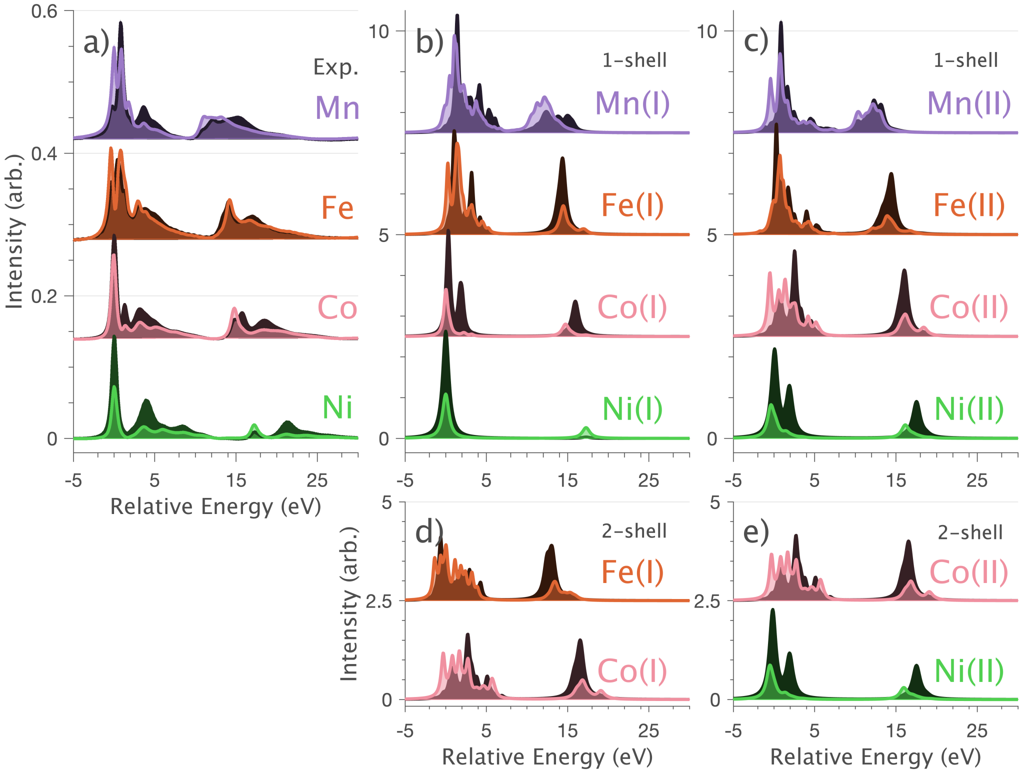

Figure S5: Comparison of normal (*light*), $\vec{E} \perp c$ and grazing (*dark*), $\vec{E}70°c$ incidence single crystal X-ray absorption spectra of $Li_2(Li_{1-x}TM_x)N$, where TM = Mn, Fe, Co and Ni to experimental (*a*) and calculated *ab initio* ligand-field multiplet calculations (*b*-*e*) at 21 K. SA-CASSCF-NEVPT2 AILFT calculations were performed on mono (*b*,*d*) and divalent (*c*,*e*) TM ions (for calculated multiplet values see Table S3). Excited state (2-shell) calculations were attempted where feasible (See S.5.1. for further details).

S.6 Ni(I) charge-transfer ligand-field multiplet dependencies

Figure S7 illustrates a systematic exploration of each individual multi-configurational ground-state of $Li_2(Li_{1-x}Ni_x)N$ through charge-transfer ligand-field multiplet theory. The high-symmetry (D_{6h}) pocket TM ions occupy within lithium nitride results in orbital degeneracies which can be considered as completely orthogonal to each other. This results in symmetry restricted orbital hybridisation and charge-transfer configurations which are virtually uncoupled to one another permitting individual optimisation of the observed high-energy satellite spectral features through $\Delta_{4s,\delta,\pi}$ and $V_{4s,\delta,\pi}$

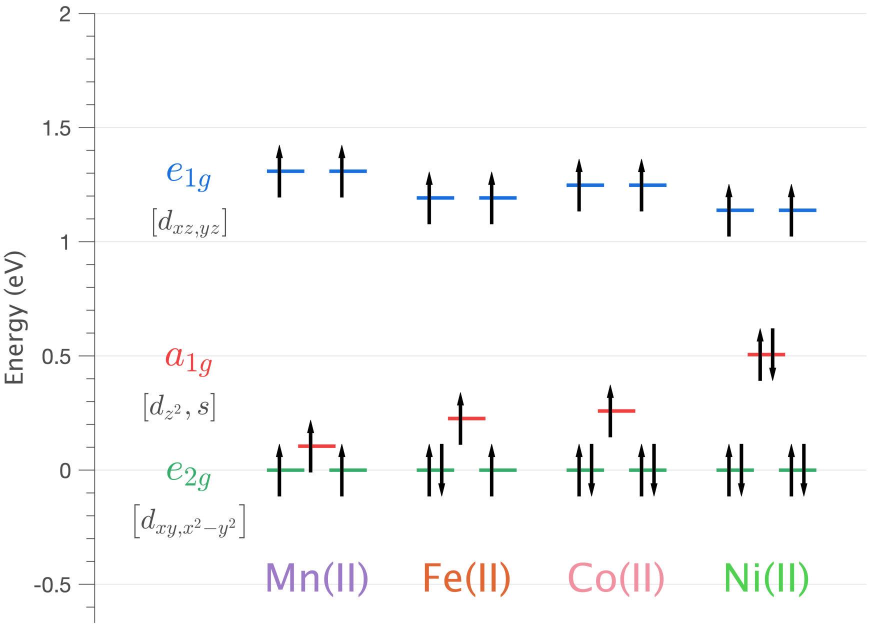

Figure S6: Energy level diagram series trend of divalent $Li_2(Li_{1-x}TM_x)N$ (where TM = Mn, Fe, Co, and Ni) calculated as one electron eigenfunctions employing a SA-CASSCF-NEVPT2 AILFT calculation.

to spectral positions and intensities. Complete tabulated values for the series as stated in Table S2.

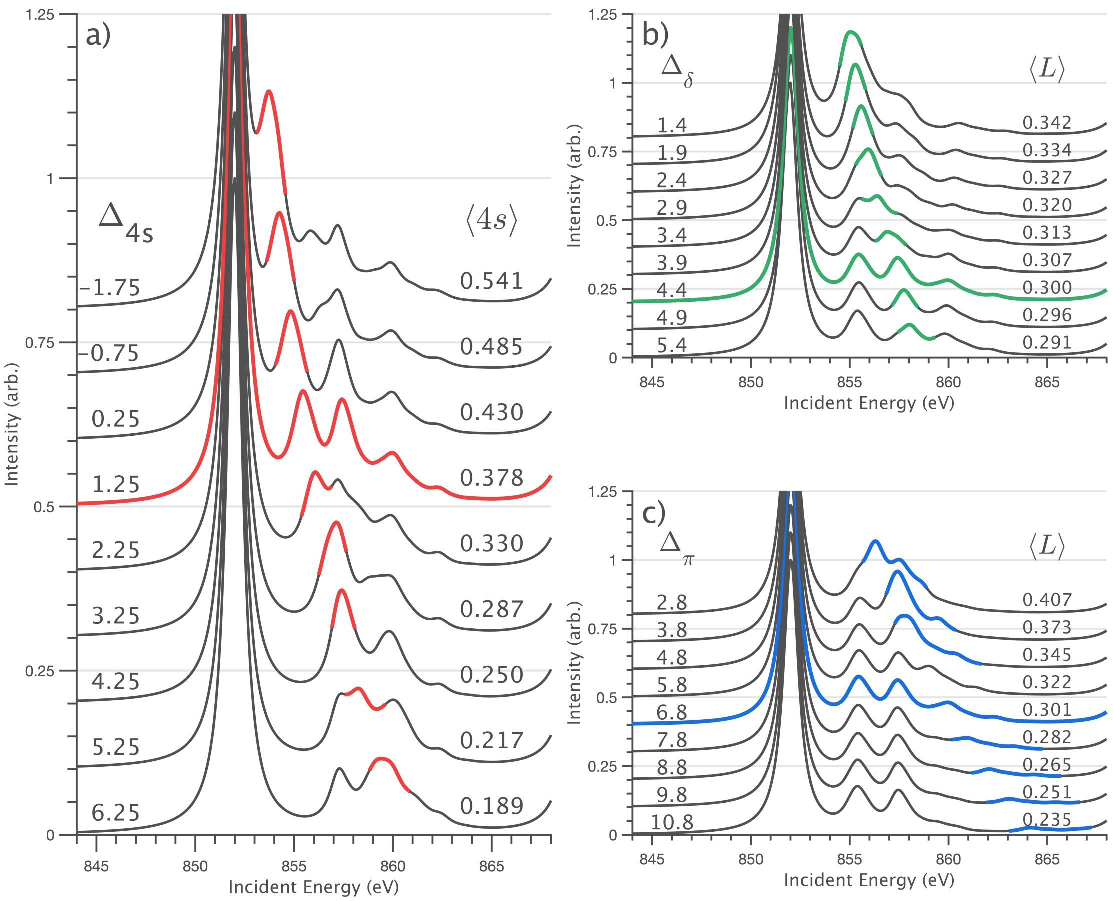

Figure S7: Systematic exploration of the multi-configurational interaction energy difference, Δ dependencies upon normal incidence ($\vec{E} \perp c$) Ni $L_{2,3}$-edge calculations. Optimised calculations in bold linewidths (Figure 6 and 7). Fixed optimised overlap, V of all calculations (as stated in Table S2) with exploration of (a) $|3d^8 4s\rangle$, (b) $|3d^8 L_\delta^-\rangle$ and (c) $|3d^8 L_\pi^-\rangle$ configuration interactions investigated with calculated expectation values of the metal $4s$ or ligand orbitals. Spectral regions marked in bold guide the eye to highlight the part of the spectrum that is most sensitive to Δ.

Table S2: Ground (GS, $3d^n$) and excited-state (ES, $2p^5 3d^{n+1}$) charge-transfer ligand-field multiplet parameters of optimised $Li_2(Li_{1-x}TM_x)N$ $L_{2,3}$-edge calculations; all values in eV. All ground- and excited-state Slater-Condon-Shortley (F^k, G^k) integrals were scaled to 80% Hartree-Fock deduced values.

	$Li_2(Li_{1-x}Ni_x)N$		$Li_2(Li_{1-x}Co_x)N$		$Li_2(Li_{1-x}Fe_x)N$		$Li_2(Li_{1-x}Mn_x)N$	
	GS	ES	GS	ES	GS	ES	GS	ES
F^2_{dd}	8.8672	9.5120	8.3440	9.0088	7.8088	8.4976	7.2576	7.9768
F^4_{dd}	5.4680	5.8744	5.1448	5.5632	4.8136	5.2472	4.4720	4.9248
F^2_{pd}		5.6784		5.2992		4.9136		4.5216
G^1_{pd}		4.2096		3.8920		3.5704		3.2448
G^3_{pd}		2.3944		2.2128		2.0296		1.8432
G^2_{ds}	0.9656	0.8584	0.9704	0.8560	0.9768	0.8569	0.9888	0.8576
G^1_{ps}		0.1472		0.1456		0.1440		0.1424
ζ_{2p}		11.509		9.750		8.530		7.121
ζ_{3d}	0.074	0.093	0.054 [a]	0.069 [a]	0.052 [a]	0.068 [b]	0.035	0.046
Ds	-0.0429	-0.0429	-0.0729	-0.0729	0.186	0.186	-0.0286	-0.0286
Dt	0.2324	0.2324	0.2683	0.2683	0.2011	0.2011	0.1789	0.1789
Δ_{4s}	1.25	2.25	0.458	1.458	1.67	2.67	1.38	2.38
V_{4s}	7.49	7.49	5.37	5.37	2.68	2.68	2.12	2.12
$\Delta_{[\delta]\pi}$	[4.40] 6.80	[5.40] 7.80	2.40	3.40	2.25	3.25	3.74	4.74
$V_{[\delta]\pi}$	[0.80] 1.80	[0.80] 1.80	1.40	1.40	0.50	0.50	0.83	0.83

[a] Scaling of 92% to atomic value as determined from EPR measurements[10].

[b] Scaling of 116% to atomic value as determined from temperature dependent X-ray absorption measurements[3].

Table S3: Mono and divalent SA-CASSCF-NEVPT2 AILFT deduced $Li_2(Li_{1-x}TM_x)N$ ground-state ($3d^n$, 1-shell) and excited-state ($3d^{n+1}$, 2-shell) multiplet parameter values. Mn(I), Mn(II) and Fe(II) excited-state multiplet parameters scaled to 80% Hartree-Fock deduced values (See Table S2) with associated TM-N deduced bond lengths. All values in eV unless stated otherwise.

	Ni(I)	Ni(II)		Co(I)		Co(II)		Fe(I)		Fe(II)	Mn(I)	Mn(II)
	GS	GS	ES	GS	ES	GS	ES	GS	ES	GS	GS	GS
F^2_{dd}	-	9.465	9.548	6.788	8.743	8.675	9.578	6.065	6.683	7.985	5.425	7.285
F^4_{dd}	-	5.982	6.227	3.644	5.702	5.468	6.246	4.101	4.358	5.182	3.627	4.832
F^2_{pd}			7.780		10.089		6.078		2.878			
G^1_{pd}			5.050		6.338		5.162		4.662			
G^3_{pd}			2.952		2.639		3.641		5.667			
ζ_{2p}			10.905		9.202		3.641		7.750			
ζ_{3d}	0.071	0.079	0.093	0.054	0.057	0.062	0.066	0.041	0.044	0.048	0.030	0.036
Ds	0.0193	-0.3071	-0.3071	0.0171	0.0171	-0.2523	-0.2523	0.0353	0.0353	-0.2349	0.0319	-0.2170
Dt	0.1584	0.1445	0.1445	0.1671	0.1671	0.1500	0.1500	0.1732	0.1732	0.1427	0.1973	0.1526
TM-N (Å)	1.8479 [b]	1.8479 [b]	1.8479 [b]	1.8100 [b]	1.8100 [a]	1.8100 [a]	1.8100 [a]	1.8737 [a]	1.8737 [a]	1.8737 [a]	1.8791 [b]	1.8791 [b]

[a] Experimentally deduced from EXAFS measurements, Co[13] and Fe[3].
[b] SCF energy minimised within the B3LYP functional.

Table S4: Ground-state expectation values of the electronic occupation of $3d$, $4s$ and ligand orbitals for $Li_2(Li_{1-x}TM_x)N$ of the optimised charge-transfer ligand-field multiplet parameters (Table S2).

	Ni	Co	Fe	Mn
$\langle d_{z^2} \rangle$	1.6151	1.6280	1.7742	1.6803
$\left\langle d_{x^2-y^2}, d_{xy} \right\rangle$	3.9631	3.8425	2.9912	1.9988
$\langle d_{xz}, d_{yz} \rangle$	2.9042	1.9528	1.9676	1.9622
$\langle 3d \rangle$	8.4823	7.4232	6.7330	5.6413
$\langle 4s \rangle$	0.3931	0.3699	0.2244	0.3170
$\langle L \rangle$	0.1246	0.2068	0.0426	0.0417

Table S5: Full-width half maximum, (FWHM) Lorentzian core-hole life time broadenings applied to calculated $L_{2,3}$-edge spectra. Gaussian FWHM consistent throughout all calculations of 0.25 eV. All values in eV.

	Cu	Ni	Co	Fe	Mn
Γ L_3, L_2	1.40, 1.85	0.55, 1.10	0.45, 1.00	0.35, 0.90	0.25, 0.80

S.7 Grazing incidence XAS and XMCD

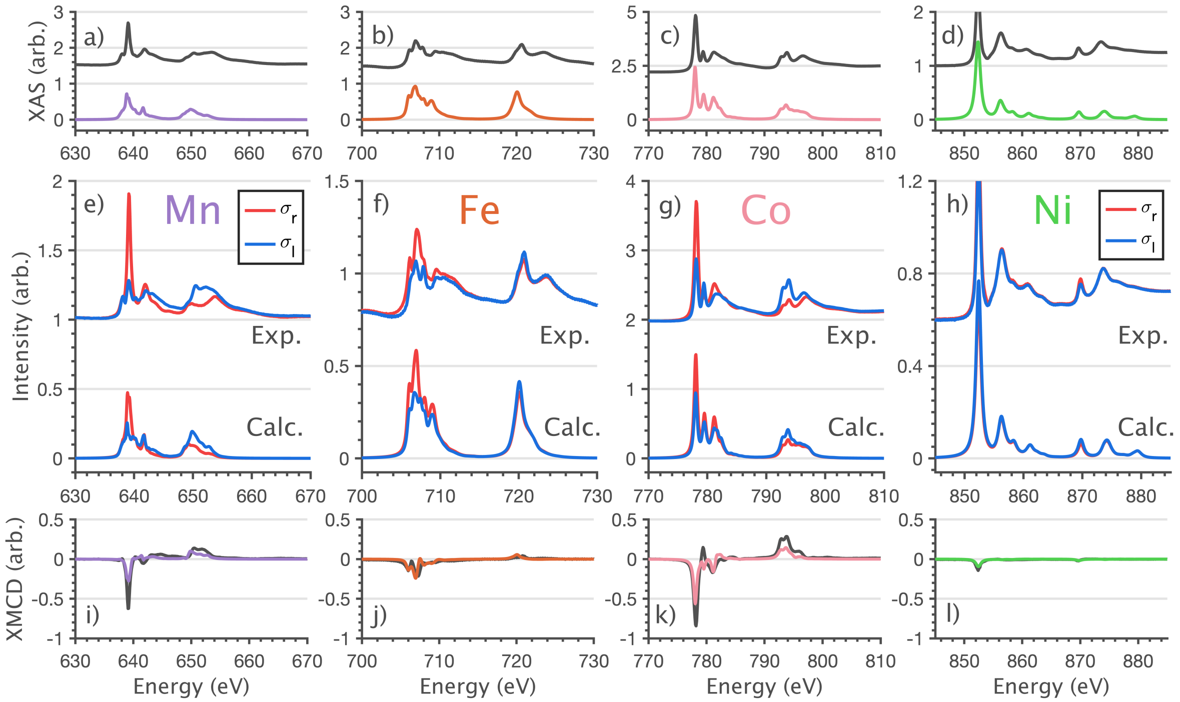

Figure S8: Grazing incidence, $\overrightarrow{E}$ 70° c, single crystal $Li_2(Li_{1-x}TM_x)N$ (where TM = Mn, Fe, Co and Ni) XAS spectra (*a*-*d*) (σ_h), XMCD spectra (*i*-*l*) (σ_r - σ_l), experimental (*black*) and optimised charge transfer multiplet calculations (*colour*), and circular polarisation absorption (*e*-*h*) (σ_r and σ_l) performed at 21 K and 14 T (H_x = 13.16 T and H_z = 4.79 T).

S.8 Zeeman Diagrams

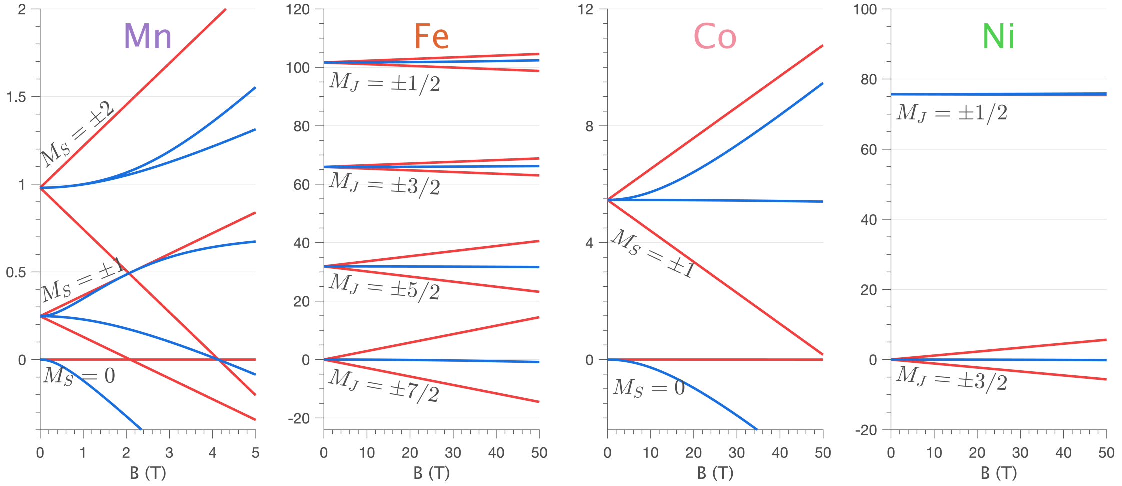

Figure S9: Calculated Zeeman diagrams for the $Li_2(Li_{1-x}TM_x)N$ series portraying the effect of a magnetic field vector on the ground-state manifold. (—) $H \parallel c$ and (—) $H \perp c$. Labelling of states as defined in Table 1.

References

[1] S. J. Clark, M. D. Segall, C. J. Pickard, P. J. Hasnip, M. I. Probert, K. Refson and M. C. Payne, *Zeitschrift fur Kristallographie*, 2005, **220**, 567–570.

[2] J. P. Perdew, A. Ruzsinszky, G. I. Csonka, O. A. Vydrov, G. E. Scuseria, L. A. Constantin, X. Zhou and K. Burke, *Physical Review Letters*, 2008, **100**, 136406–136406.

[3] M. S. Huzan, M. Fix, M. Aramini, P. Bencok, J. F. W. Mosselmans, S. Hayama, F. A. Breitner, L. B. Gee, C. J. Titus, M. A. Arrio, A. Jesche and M. L. Baker, *Chemical Science*, 2020, **11**, 11801–11810.

[4] S. P. Gao, C. J. Pickard, M. C. Payne, J. Zhu and J. Yuan, *Physical Review B - Condensed Matter and Materials Physics*, 2008, **77**, 115122.

[5] V. I. Anisimov, J. Zaanen and O. K. Andersen, *Physical Review B*, 1991, **44**, 943–954.

[6] M. Cococcioni and S. De Gironcoli, *Physical Review B - Condensed Matter and Materials Physics*, 2005, **71**, 035105.

[7] M. W. Haverkort, M. Zwierzycki and O. K. Andersen, *Physical Review B*, 2012, **85**, 165113.

[8] M. W. Haverkort, Y. Lu, R. Green, S. Macke, M. Retegan, M. Brass and S. Heinze, *Quanty v0.7b*, 2022, `quanty.org`.

[9] M. Retegan, *Crispy: v0.7.3*, 2019.

[10] C. Albert, T. J. Ballé, F. A. Breitner, Y. Krupskaya, A. Alfonsov, Z. Zangeneh, S. Avdoshenko, M. S. Eldeeb, L. Hozoi, A. Vilangottunjalil, E. Haubold, A. Charnukha, B. Büchner, A. Jesche and V. Kataev, *Inorganic Chemistry*, 2021, **60**, 4497–4507.

[11] R. K. Hocking, E. C. Wasinger, F. M. De Groot, K. O. Hodgson, B. Hedman and E. I. Solomon, *Journal of the American Chemical Society*, 2006, **128**, 10442–10451.

[12] E. C. Wasinger, F. M. De Groot, B. Hedman, K. O. Hodgson and E. I. Solomon, *Journal of the American Chemical Society*, 2003, **125**, 12894–12906.

[13] D. Muller-Bouvet, J. P. Pereira-Ramos, S. Bach, P. Willmann and A. Michalowicz, *Inorganic Chemistry*, 2014, **53**, 6127–6131.
